# Novel nontarget LC-HRMS-based approaches for evaluation of drinking water treatment

**DOI:** 10.1007/s10661-023-11348-w

**Published:** 2023-05-26

**Authors:** Petra Nováková, Helena Švecová, Adam Bořík, Roman Grabic

**Affiliations:** Faculty of Fisheries and Protection of Waters, South Bohemian Research Centre of Aquaculture and Biodiversity of Hydrocenoses, University of South Bohemia in České Budějovice, Zátiší 728/II, 389 25 Vodňany, Czech Republic

**Keywords:** Organic micropollutants, Drinking water treatment, Nontarget analysis, Removal efficiency, Treatment effect, Log2FoldChange

## Abstract

A conventional evaluation methodology for drinking water pollution focuses on analysing hundreds of compounds, usually by liquid chromatography–tandem mass spectrometry. High-resolution mass spectrometry allows comprehensive evaluation of all detected signals (compounds) based on their elemental composition, intensity, and numbers. We combined target analysis of 192 emerging micropollutants with nontarget (NT) full-scan/MS/MS methods to describe the impact of treatment steps in detail and assess drinking water treatment efficiency without compound identification. The removal efficiency based on target analytes ranged from − 143 to 97%, depending on the treatment section, technologies, and season. The same effect calculated for all signals detected in raw water by the NT method ranged between 19 and 65%. Ozonation increased the removal of micropollutants from the raw water but simultaneously caused the formation of new compounds. Moreover, ozonation byproducts showed higher persistence than products formed during other types of treatment. We evaluated chlorinated and brominated organics detected by specific isotopic patterns within the developed workflow. These compounds indicated anthropogenic raw water pollution but also potential treatment byproducts. We could match some of these compounds with libraries available in the software. We can conclude that passive sampling combined with nontargeted analysis shows to be a promising approach for water treatment control, especially for long-term monitoring of changes in technology lines because passive sampling dramatically reduces the number of samples and provides time-weighted average information for 2 to 4 weeks.

**Supplementary Information:**

The online version contains supplementary material available at 10.1007/s10661-023-11348-w.

## Introduction

Threatening water quality by chemical compounds from human activities represents a severe worldwide problem with rising risks, especially in the consequences of climate change. Increasing numbers and quantities of organic pollutants, like pharmaceutical and personal care products, pesticides, surfactants, phthalates, and artificial sweeteners, continuously enter the aquatic environment (Galindo-Miranda et al., 2019; Hartmann et al., 2018; Tröger et al., 2018). These chemical micropollutants (MPs), which occur in water bodies at concentrations ranging from pg/L to ng/L, may migrate to various environmental compartments and endanger living organisms (Bletsou et al., 2015; Gavrilescu et al., 2015; Schwarzenbach et al., 2006).

Several sources of known or unknown organic MPs — untreated or treated wastewater (Hernández et al., 2015; Kim & Zoh, 2016; Yang et al., 2017), agriculture, or industry, contribute individually or as complex to water resource threats (Peng et al., 2018; Ternes et al., 2015). Drinking water treatment processes are usually designed for conventional pollutant removal. The rising number and concentration of chemical compounds with highly varying physical–chemical properties can be problematic for treatment success and consequently threaten the quality of drinking water (Galindo-Miranda et al., 2019; Reemtsma et al., 2016; Schwarzenbach et al., 2006). Advanced treatment steps seem to be a significant improvement, especially for polluted water sources (Rodriguez-Narvaez et al., 2017; Teodosiu et al., 2018; Tröger et al., 2020). Organic micropollutants can be harmful in their parent form or after abiotic or metabolic transformation (Bletsou et al., 2015; Sousa et al., 2018). Water treatment processes such as ozonation and chlorination lead to the presence of the transformation products (TPs) into outlets even when parent substances have been removed. TPs’ toxicity or soil/water mobility properties can significantly differ from parent compounds, and their detection and identification require new analytical methods (Bletsou et al., 2015). Ozone is applied as a disinfection step in drinking water treatment and causes oxidation of compounds dissolved in water. Even in the case of complete dissipation of target compounds, totally unknown products can be formed with potentially higher toxicity and lower removal disposition (Brunner et al., 2019). Another broadly used disinfection process is chlorination, also suspected of forming halogenated TPs (Zahn et al., 2016) and disinfection byproducts of different chemical classes (Yang et al., 2017).

A conventional water quality assurance approach for trace organics is anchored in the legislature. It is based on targeted analytical methods by liquid chromatography hyphenated with mass spectrometry (LC–MS) (Brack et al., 2017), even though target monitoring cannot provide a complex overview of water contamination because thousands of micropollutants can be presented (Altenburger et al., 2015; Gosetti et al., 2016). High-resolution mass spectrometry (HRMS) operated in full-scan mode increases the number of revealed MPs in water matrices. In combination with the data-independent acquisition (DIA), relatively selective MS/MS spectra are provided for MP structural confirmation. Although the DIA provided higher selectivity compared to, e.g. all ion fragmentation, the selectivity is mainly affected by precursors isolation window width, which is a compromising factor compared to data-dependent acquisition (DDA). Product spectra are associated with individual precursors. The benefit of DIA against DDA is a vast number of fragmented productions without precursor preselection based on intensity or mass. Consequently, false negatives can be reduced because lower-intensity ions are not discriminated (Zhou et al., 2017). HRMS-enhanced selectivity enables screening without previous information about compounds in a sample. Nontarget screening (NTS) brings a comprehensive investigation of micropollutants (Bletsou et al., 2015; Brack et al., 2019; Krauss et al., 2010; Schymanski et al., 2015), reveals the maximum number of MPs and their fate in water treatment (Hollender et al., 2019; Sousa et al., 2018), and identifies unknown MPs or fragments, (Brunner et al., 2019; Hollender et al., 2017; Nürenberg et al., 2019). The HRMS data set from the recent interlaboratory study on passive drinking water sampling showed that participants’ in-house methods were more successful in detecting internal standards than the predefined mandatory method (Schulze et al., 2021). Also, a mass error was observed within 12 ppm independently of the instrumentation used for the analysis. This study confirms the general applicability of HRMS independently of the instrumentation and method used for data collection.

The nontarget analysis applied to water treatment offers several directions in dataset processing. All features (results from the processing procedure with exact *m/z* ratio and retention time) per sample can be used for pollution visualization or removal efficiency (RE) evaluation (Nürenberg et al., 2015). The workflows and evaluation of voluminous HRMS data offer a comparison of samples or treatment processes by the compounds detected in full-scan spectra (Müller et al., 2011; Nürenberg et al., 2019). Basically, we can divide the methods into those using raw files processed by commercial software (often limited to one vendor data format) and those using software processing exported data, e.g. open access MZmine (Pluskal et al., 2010). The methodology and workflows for studying different aspects of treatment processes in wastewater or drinking water production were reported in a few recent publications summarized in SM1 Table S1. Micropollutant removal value calculated from target compound concentration before and after treatment is usually applied to rate drinking water treatment plants (DWTPs). Contrary, NTS does not provide quantitative data, only a list of detected signals and their intensity as peak areas. However, these properties can also be used to comprehensively evaluate treatment effects considering the whole sample load and compound changes along the technology line.

In the presented study, we aimed to prove a comprehensive approach to evaluate water treatment technology. Our article brought novelty by combining field samples taken on different treatment stages, and a broad range of target analyses and nontarget screening was integrated. Sampling points were situated at inlets, inside treatment (before granulated active carbon filters (GAC)), and outlets to understand the treatment steps’ influence and reveal potentially formed compounds. We focused on passive sampling (polar organic chemical integrative samplers (POCIS)) for their integrative sampling ability over a defined period, while grab water samples can be affected by unequal pollutants distribution in water flow (Vrana et al., 2021). Formed compounds were characterized and reviewed separately. The nontarget approach can confirm the hypothesis that MP composition and concentration are highly affected by water levels fluctuating during the year and water source flow (Golovko et al., 2014; Švecová et al., 2021). Therefore, the interpretation of results was expanded by comparing raw water quality, season impact, and the effect of treatment steps.

## Materials and methods

### Chemicals and solvents

Methanol, toluene, dichloromethane used as extractions solvents, and LC mobile phases were purchased in LC–MS grade quality (Merck, Darmstadt, Germany), the same as additives in mobile phases like formic acid (Merck) and ammonium acetate (Merck). Ultrapure water was obtained from an Aqua-MAX-Ultra system (Younglin, Korea). Information about used native and isotope-labelled reference standards is listed in SM2 Table S1. Working standard mixtures were prepared by dissolving standards in methanol and diluting them to 1 µg/mL concentration.

### Sampling sites

Due to generally low contamination load and further transformation during water retention, water reservoirs outside urbanization are considered appropriate resources. However, river water flowing through urbanized areas is often used in a shortage of other resources. Surface water is affected by human activities, agriculture, WWTPs outflows, and river flow fluctuation (Švecová et al., 2021). There is no possibility of controlling the raw water quality compared to reservoirs.

**Fig. 1:**
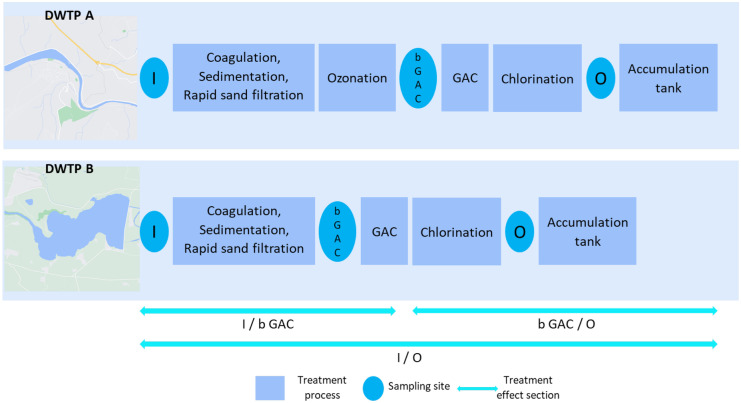
Schema of DWTP and B with sampling sites (I, inlet; b GAC, before granular active carbon filters; O, outlet) and treatment section used for treatment effect evaluation

This study examined two drinking water treatment plants in the Czech Republic, detailed in SM1 Table S2. Besides conventional treatment, both DWTPs have filters with granular activated carbon (GAC), and DWTP A uses ozonation as an advanced oxidation process. The water source for DWTP A is the river, and DWTP B is a water reservoir in a rural area. The schema of sampling points (inlet, before granulated active carbon filters (GAC)), outlet) and the treatment section is visualized in Fig. 1. Water source quality partly determines the level of final drinking water.

We sampled DWTPs in the spring and autumn of 2019 within this study. Sampling sites were situated at the inlet, outlet, and before GAC filters at each DWTP (see list of sampling points in SM1 Table S3). During the field study, operational/technical issues appeared. In the spring season, ozonation step at DWTP A was disabled because of building reconstruction. We found this unexpected event as an opportunity to examine efficiency with the same technology but without the oxidation step. During autumn sampling at the same DWTP, an upstream accident with leakage of wood impregnation substances to the river happened. For 2 days, raw water was taken from another river. We added one subsequent POCIS set at this treatment plant, and the second set of samples was also included in the study. All those events document difficulties in planning and performing the study at two remote locations. However, such field studies show more realistic scenarios of routine operations of DWTPs. These facts are addressed in the ‘Results and discussion’ section. Detailed information about sampling is also given in SM1 Table S3.

### Sampling and sample preparation

We used commercially available polar organic chemical integrative samplers in pesticide-specific configuration (Nya Exposmeter AB, Tavelsjö, Sweden). These flat disc-shaped passive samplers consist of solid triphasic admixture (Isolute ENV + Carboxen 572 dispersed on S-X3 Bio Beads) filled between two microporous polyethersulfone membranes (0.1 µm pores). Passive samplers were placed in protective metallic holders and deployed in water for 14 days. Exposed samplers were cleaned with ultrapure water and, after transport on ice, stored at − 20 °C until extraction. Standardized extraction procedure with a solvent mixture — methanol:toluene:dichloromethane (1:1:8 v.v.) — was used (Alvarez et al., 2005). This procedure was recently validated for a wide range of pharmaceuticals in our laboratory (Vrana et al., 2021). Extracts were concentrated using rotary vacuum evaporator G1 (Heidolph, Germany) to 1 ml. Extracts were stored in preweighted vials at − 20 °C. Aliquots of 250 µL were diluted with 250 µL of ultrapure water, spiked with isotope-labelled standards mix (2.5 ng), and analysed. The laboratory blank sample was prepared the same way by extracting clean POCIS from the same batch.

### HPLC–ESI–MS/MS target analysis

The target analysis was carried out by TSQ Quantiva Triple-Stage Quadrupole MS (Thermo Fisher Scientific) using reversed-phase chromatography separation at Hypersil GOLD aQ column (2.1 mm × 50 mm, particle size 5 μm, Thermo Fisher Scientific). The injection volume was 10 μL of the sample extract. The routine method was based on the previous work of Grabic et al., 2012, extended by Švecová et al., 2021 and Vrana et al., 2021 in their previous papers. A list of target analytes with details and *m/z* transitions is attached in SM2 Table S1, and information about HPLC–MS/MS methods set is in SM2 Table S2. The MS/MS data was quantified using internal standards and the matrix matching standard method.

### HPLC-ESI-(HR)MS nontarget analysis

POCIS extracts were analysed by injecting 10 μL of the sample by autosampler (HTS XT-CTC autosampler, CTC Analytics AG, Zwingen, Switzerland) into high-performance liquid chromatography (HPLC) system, Accela 1250 LC pump (Thermo Fisher Scientific, San Jose, CA, USA). Reversed-phase LC chromatography at Kinetex EVO C18 (2.1 mm × 50 mm, particle size 5 μm, Phenomenex, Torrance, CA, USA) was used for separation using the methanol gradient in water (both phases buffered with 10 mmol NH4Ac and 0.1% formic acid). The mobile phase composition, gradient, and other method details are described in SM2 Table S2. The hybrid quadrupole-orbital trap HRMS Q-Exactive (Thermo Fisher Scientific) was used for compound detection. Two separate runs were performed in positive and negative electrospray ionization (ESI +), and (ESI-) modes. The ESI parameters were set as follows: 3.5/2.8 kV ionization voltage for ± mode; 40 arbitrary units of sheath gas; 10 arbitrary units of auxiliary gas; vaporizer temperature 275 °C,;and capillary temperature 300 °C.

Combined full-scan and MS/MS experiments were used to detect present compounds. HRMS data were acquired with the following parameters: the resolution of 70,000 FWHM; scan range 100–1000 m*/z*; automatic gain control 3.10^6^; and maximum filling time of 50 ms. MS/MS experiment was performed in data-independent acquisition (DIA) mode for fragmentation of all precursors from a defined range of *m/z* (isolation window 110 m*/z* for inclusion list of *m/z* 150, 250, 350 450, and isolation window 500 for *m/z* 750, stepped normalized collision energy 15, 35, 55, automatic gain control 1.10^6^, and maximum filling time 30 ms).

### Data processing and analysis

The MS/MS data from target analysis were quantified by software Trace Finder 3.3 (Thermo Fisher Scientific) expressed as ng/POCIS for three benzotriazole derivates, 81 pharmaceuticals, and 98 pesticides (concentration and limits of quantification in SM1 Table S4). Calculating of summary removal efficiency was based on quantified MPs concentration (c) in inlet and outlet (RE = (*c*_Inlet_ – *c*_Outlet_) / *c*_Inlet_) × 100. Result visualization with PCA was performed in the STATISTICA v.12 software for Windows (StatSoft, Czech Republic).

LC-HRMS full-scan DIA data was processed by Compounds Discoverer 3.1 software (CD 3.1, Thermo Fisher Scientific). The workflow was assembled to detect micropollutants automatically and suppress background contamination or low-quality signals. Raw files were directly uploaded into software and handled with consequent steps for alignment of chromatograms, peak picking, background signals subtraction, molecular formula assignment, isotope pattern scoring, differential analysis, and searching online databases based on a predicted formula and grouping compounds across samples. Mass tolerance for peak detection and formula prediction was set to five ppm, RT alignment to 0.3 min, and intensity threshold to 2000. Compounds with isotope patterns were searched by this list: Cl, Cl2, Cl3, Cl4, Cl5, Cl6, Cl7, Cl8, Br, Br2, Br3, Br4, Br5, ClBr, ClBr2, and Cl2Br. Detailed properties, settings, and parameters for positive and negative modes are listed in SM2 Table S3.

Datasets from positive and negative mode analysis were handled individually with different workflow and reported together in results. The software created the list of features (all detected signals with *m/z*, deduced molecular weight, signal intensity, and retention time). It grouped them by molecular weight and retention time (RT) into the list of compounds for each sample. All possible background contamination was removed by comparing the blank sample and extract signals (sample/blank ratio < 5).

After peak detection, alignment, and compound prediction, the software implements differential analysis. Compound features were compared comprehensively against each other according to the postulated hypothesis. Samples from inlets and before GAC were put against samples from outlets to characterize and visualize changes coming from treatment steps. Outputs are statistical data about every compound found in samples (ratio, log2FoldChange) or overall pollution (compound number, sum of peak areas, principal compound analysis). Results from the differential analysis are directly available in software visualization by datasets or figures.

### Evaluation of treatment effect

The number of compounds detected after the corresponding treatment step is the most straightforward efficiency estimation. Nontarget analysis precludes routine removal efficiency because signal intensities as peak area may vary over orders of magnitude for different compounds at the same concentration level. Consequently, the treatment effect (TE) was calculated in two ways: from detected peak numbers or peak areas of features with the same formula and retention time. For the investigation of changes along the treatment steps, log2 fold change (log2FC) values were calculated from peak areas for each compound by CD 3.1. as a part of the differential analysis. It is the ratio of value in input and output (start and end of treatment step) in a logarithmic scale to gain results from negative to positive values. The categories for groups differentiated by log2FC values were adopted from the methodological study by Bader et al. (2017). The formula is given as follows:log2FC = log2 (area before treatment / area after treatment)

The difference between the compound areas in the sample before and after treatment indicates a level of change. When the value of log2FC is > 1 or <  − 1, the corresponding signal decreases or increases, respectively, and values between − 1 and + 1 are considered insignificant changes. This categorization was evaluated and confirmed with an analysis of the relative standard deviation (RSD) of isotope-labelled standards peak areas, which was lower than 20%. Also, the matrix effect can cause deflection in this range from zero. Eliminated compounds have log2FC level + Infinity (found only at the start of the treatment section), and − Infinity level belongs to the formed compounds detected only at the end of the treatment section. The treatment process was divided into three sections according to sampling sites: inlet/outlet (I/O), inlet/before GAC (I/b GAC), and before GAC/outlet (b GAC/O), for a better understanding of which part affects compounds more.

## Results and discussion

### Concentrations of analysed target micropollutants across treatment plants, seasons, and technology lines

The broad spectrum of 182 emerging micropollutants (three benzotriazoles, 98 pesticides and metabolites, 81 pharmaceuticals, and metabolites) was included in the target analysis. Quantified amounts of target compounds are listed in SM1 Table S4, and calculated removal efficiencies for each treatment section of both DWTP in two seasons are reported in SM1 Table S5. The differences among the DWTPS, seasons, and sampling sites are seen in the data. However, the data set’s complexity needed the application of exploratory statistics — principal component analysis (PCA).

**Fig. 2:**
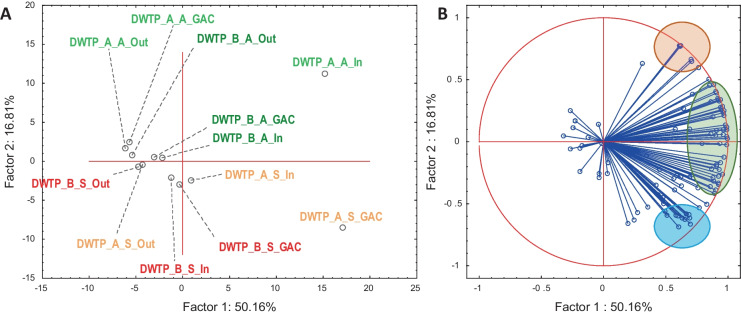
PC1 vs. PC2 plane plot of cases (**A**) and variables (**B**) of principal component analysis of the quantified micropollutants in DWTPS across DWTPS, treatment, and seasons. Green labels in **A** visualize autumn sampling and red spring ones — light tunes of the corresponding colour show DWTP A and dark ones DWTP B. Coloured circles in **B** indicate markers of seasonality (red and blue) and highly contaminated water (green)

Two PC explained 67% of the data variability. The plot of the cases (sampling events) and variable projection on the PC1 and PC2 plane plots is shown in Fig. 2A. PC1 correlates with the total concentration of compounds in POCIS. The highest micropollutant levels were found in DWTP A using river water as a resource. Telmisartan, tramadol, benzotriazoles, irbesartan, clarithromycin, and lamotrigine were found at high levels in DWTP A, which indicated communal pollution of the resource water. Surprisingly, the level before GAC was about twice higher than in the inlet in spring sampling. It could be caused by the absence of ozonation and the fact that conventional treatment can mobilize compounds (Ren et al., 2020). DWTP B showed a much lower pollution level because of the resource reservoir location, as is seen in the position of both DWTP B inlet samples in Fig. 2A. Anyhow, all outlets (drinking water) showed the lowest MP levels together with the site prior to the GAC filter in DWTP A, in autumn, when ozonation was on.

The PC2 can be attributed to the seasonal difference in river water quality in DWTP A. We have already described the seasonal differences caused by different flows in spring and autumn (Švecová et al., 2021). The red circle in Fig. 2B indicates compounds related to autumn pollution. This group contains some pharmaceuticals, e.g. venlafaxine and its metabolite O-desmethyl venlafaxine, and low levels of fungicides appearing only in autumn (clomazone, azoxystrobin, cyproconazole, triticonazole, etc.). Markers of spring MPs profile can be characterized as currently used pesticides and their metabolites (metolachlor ethane sulfonic acid, metazachlor ethane sulfonic acid, atrazine 2-hydroxy, acetochlor oxanilic acid, alachlor oxanilic acid, diazinon, etc.). Due to the dam’s retention time, the seasonal difference is more critical for raw river water than reservoirs.

### The removal efficiency by target analyses

Conventionally, the removal efficiency was calculated for each compound present at least in one sample in the corresponding sampling time and the location above LOQ. The LOQ value was used for the calculation as the worst-case scenario. The results are reported in SM1 Table S5. There are apparent differences in median RE for all compounds in the group based on inlet/outlet concentrations among the groups of MPs, DWTPs, and seasons. Benzotriazoles are removed between 91 and 100%, pharmaceuticals from 55 to 92%, and pesticides from 19 to 82%. The absence of ozonation causes the highest difference in the case of polluted river water in DWTP A. Pesticides and their metabolites are more efficiently removed only when ozonation in combination with GAC is applied.

**Fig. 3:**
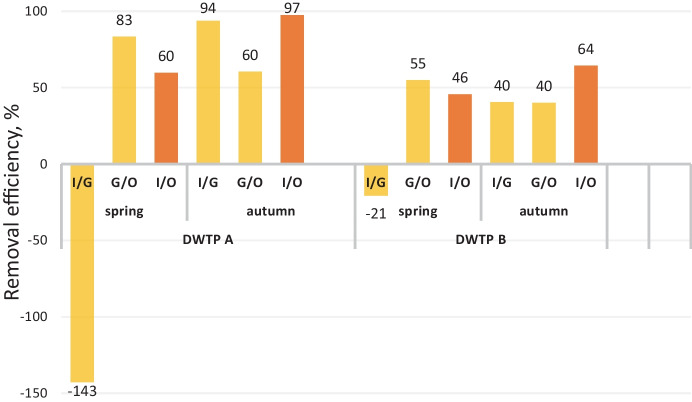
Calculated removal efficiency (in per cent) of 182 target analytes in the whole DWTP process (inlet/outlet in orange colour) and in two treatment sections (inlet/before GAC and before GAC/outlet in yellow colour)

The above-mentioned results are too complex and challenging to compare with nontargeted data. We simplified the evaluation by using the sum of concentrations of all compounds. The results of removal efficiencies for each technology step are visualized in Fig. 3. RE results in each treatment section varied depending on processes; e.g. the first part of treatment is not proposed for micropollutants elimination, and even concentration increase was observed. A negative value means a higher concentration was found after treatment than at the start. This observation is common in wastewater treatment and can be explained by transformation, desorption, or breakthrough from GAC filters (Ren et al., 2020). There is a main difference between the DWTP A in spring without ozonation and autumn results with ozonation. The worst RE − 142% showed high pollution of raw water and ineffective first (conventional) treatment when MPs could be mobilized from the matrix until eliminated by GAC. Aggregate RE for DWTP A in spring was 60%. However, the same plant in autumn reported improvement by functional ozonation step before GAC, which increased RE in the first section to 94%, and total RE was 97%. The advantages of ozonation for micropollutants elimination were already mentioned (Petrović et al., 2003), together with unintended consequences in the formation of transformation products.

DWTP B indicates approximately similar RE in both seasons and across all sections except the first section in spring with a negative value. Although the pollution load of source water for DWTP B was estimated and confirmed as lower, removal efficiency was only about 50%. This finding illustrated that only active carbon filters did not sufficiently enough remove MPs, and advanced treatment methods are required. DWTP B’s efficiency seems to correspond with the study of 13 DWTPs in Europe and Asia, where RE was, on average, 65% (Tröger et al., 2021). The ageing of GAC filters is the main factor causing efficiency regression, as was shown in Tröger et al. (2018).

### Nontarget analysis and matrix effect validation

We evaluated a treatment effect based on signal intensity changes for each detected compound. Matrix effects like signal suppression or enhancement can influence peak areas and modify results (Schulze et al., 2020; Vrana et al., 2021). The response of isotope-labelled standards (IS) was used to validate the method. A mixture of standards was spiked into all samples and blanks after extraction. The concentration level of the spike was 2.5 ng in 250 uL of POCIS extract. Peak areas were quantified via the software Trace Finder 3.1 (Thermo Fisher Scientific). Signal intensity shifts caused by matrix effects were established by calculating the relative standard deviation (%RSD) in chromatograms of positive and negative ionization modes. Maximum and minimum values of areas are listed in SM2 Table S4. The calculated RSD for all isotope-labelled standards did not exceed 20%. Equally, the matrix effect on retention time was evaluated with the highest RSD of 6%.

### Compound list

Software calculation of elemental composition from molecular weight (MW) and isotopic patterns for each feature are the keys for tentative compound formula prediction and subsequent match status annotations from search databases. This way, features are processed to the compounds list, which is consequently interpreted by several evaluation strategies. Filtering out of compounds of interest (halogenated one or with an intensity ratio of our interest) enables a comprehensive comparison of samples without exact compound identification.

The first necessary step to obtain results was to distinguish features and compounds. Grouping of features found in samples was done by molecular mass and RT. The resulting compounds list was consequently filtered with the following criteria — an area greater than 50,000, RT greater than 1 min, and the predicted elemental composition (formula). Detected features in extracts were filtered from 22,931 to 2881 compounds for ESI + and 9052 to 1689 compounds for ESI-. In SM1 Table S6 reports compounds grouped by sampling points with their summed numbers and peak areas.

### Raw water quality

Overall pollution load in natural water sources can be considered by the numbers of detected compounds and their areas from the nontarget analysis. Variability of results at inlets to DWTPs in the spring and autumn can be linked with water flow and temperature. The difference between seasons was approximately two-time higher water flow in spring (DWTP A 4.32 m^3^/s and DWTP B 2.72 m^3^/s) than in autumn (DWTP A 2.72 m^3^/s and DWTP B 0.88 m^3^/s). The inlet’s NTS results correspond to the presumption that the water reservoir for DWTP B is less affected by pollution than the river supplying DWTP A and to the results of the target analyses. According to compound number (DWTP A spring 1 003, autumn 1 275; DWTP B spring 719, autumn 722) and total peaks areas (DWTP A spring 1.6.10^10^, autumn 2.5.10^10^; DWTP B spring 1.4.10^10^, autumn 1.3.10^10^), raw water quality is more stable in the rural reservoir than in the downstream stretch of the river. There were found fewer compounds in both seasons and only low seasonal change. In addition, DWTP B always uses the best raw water layer in the reservoir to minimize the load of the impurities as particles and dissolved metals such as Fe and Mn, which can affect basic treatment technology.

**Fig. 4:**
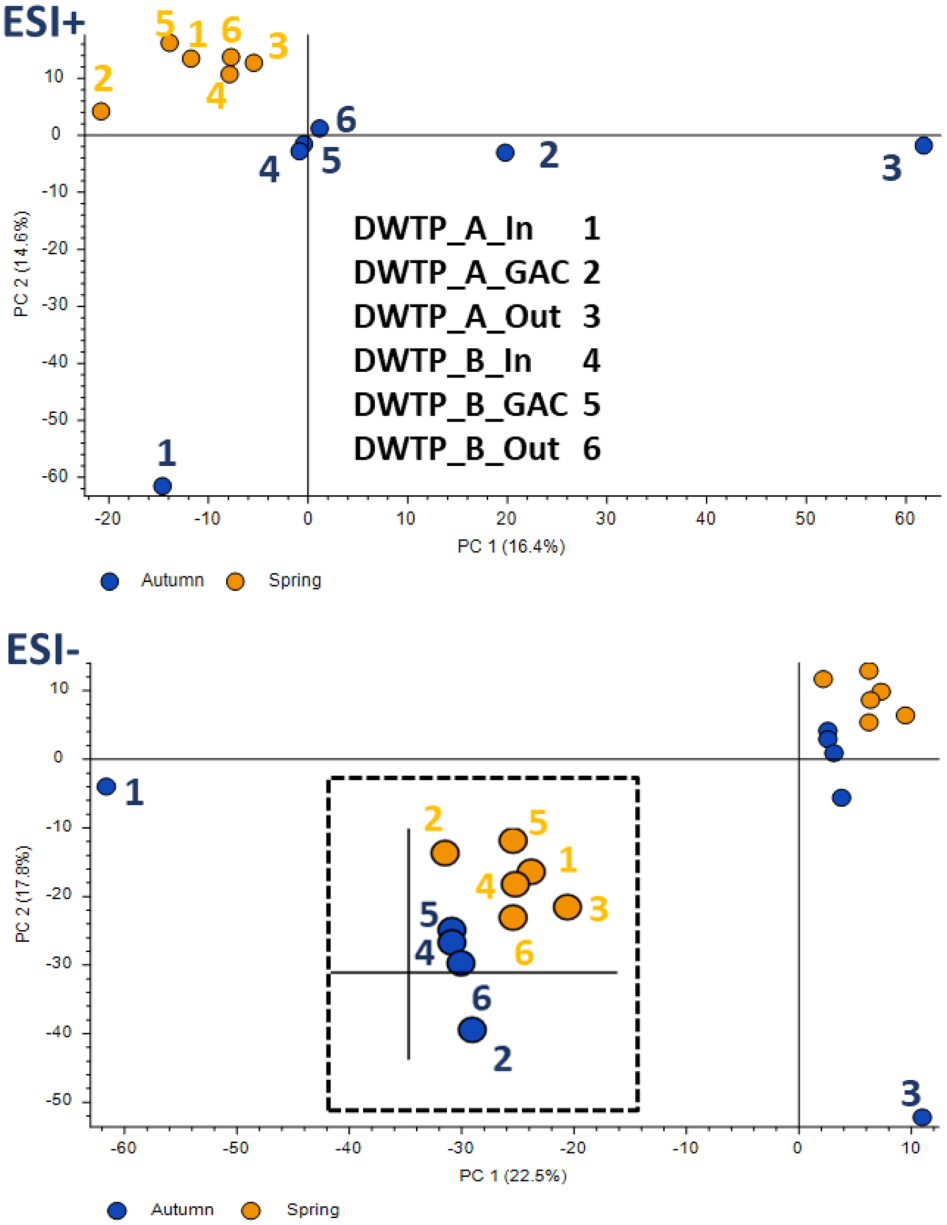
Principal component analysis (PCA) for ESI + (above) and ESI- (below) from the nontargeted analysis. Autumn sampling is coloured dark blue and spring one in orange points and labels. The dashed picture in the middle of the ESI- PCA plot is a zoom of the upper right corner

According to our previous work (Švecová et al., 2021), compounds present in raw river water with the seasonal changed flow were raised in autumn, probably affected by lower river flow, and consequently lower dilution. This statement is supported by NTS’s principal component analysis in Fig. 4. PC1 and PC2 seemed to correlate with the pollution level and seasonality, similarly to PCA analysis of target data. However, PC1 and PC2 explained only about 30% of variations in ESI + and 39% in ESI- of nontarget results. The samples’ distances and sampling events’ grouping also show a different picture. The samples from spring sampling are separated, independently on DWTP, from the autumn ones. Ozonation has a much higher impact, shown in NTS results, than in target analysis data (Fig. 2). This finding supports our assumption that targeted analysis cannot provide insight into the complex pollution of raw water and the potential formation of treatment byproducts. In both NTS PCA plots, autumn sampling sites at DWTP A (especially inlet and outlet) lay far away from other samples. Water temperature in both seasons (autumn average 10.1 °C, spring average 8.6 °C) was not significantly different to discuss the effect of temperature.

### Nontarget evaluation of treatment

**Fig. 5:**
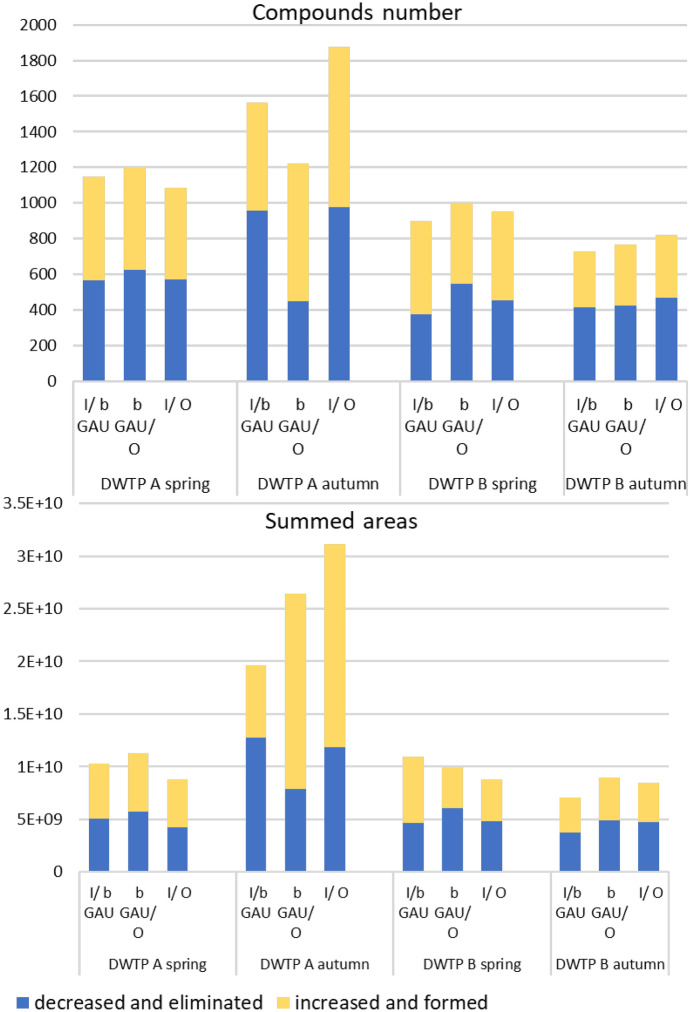
Trends of treatment effects while comparing positive (decreased and eliminated in blue colour) and negative (increased and formed in yellow colour) changes

The data were processed according to the procedure given in the material and methods chapter. In the first step, all compounds from positive and negative ESI modes were divided by sampling sites (visualization in Fig. 5). Summarized compound numbers and peak areas have similar trends for both approaches except for DWTP A in autumn. The ozonation step (missing in spring) impacted the formation of new chemicals/transformation products in autumn. Formed/changed compounds caused the increase of numbers and even more total peak area found in final water (all values listed in SM1 Table S7). NT screening quickly revealed an elementary aspect of unknown compounds’ fate in treatment and the weakness of target compound monitoring. A more illustrative evaluation of these trends can be performed by separating the compound list into groups according to their fate during treatment.

#### Compound numbers and peak area changes

The compound numbers and summed areas for each log2FC level are reported in SM1 Table S7 as indicators of changes in the treatment technology. The categories for groups differentiated by log2FC values were adopted from Bader et al. (2017). Table 1 shows that compounds eliminated and formed during treatment are almost equal. The results for individual treatment steps and DWTPS are visualized as eliminated and decreased versus formed and increased compounds in Fig. 5. Again, both groups showed a similar trend. This approach seems beneficial for quickly evaluating compounds’ fate in treatment. There is a clear unbalance between decreased and eliminated compounds, caused by the fact that compounds can be filtered out by minimal peak area setting in the workflow and consequently counted as eliminated. The same happened with increased and formed compounds. This step helps to reveal why calculating overall RE from NT analysis is not too valid. A few publications mentioned and applied a similar approach for effectivity assessment in WWTPs study (Itzel et al., 2020; Nürenberg et al., 2015; Schollée et al., 2018). Also, Nürenberg et al. (2015) presented the growth of transformation products in WWTP effluents identified via NT workflow, corresponding to our results. Although wastewater treatment methods and pollution levels differ from drinking water, more complex analyses benefit both technologies. Bader et al. (2017) presented a method for comparing the ozonation effect with fold change values for drinking water processes. However, our case study allowed us to monitor consequent treatment and compound fates.

**Table 1:** Numbers of compounds with log2FC value levels for both seasons from all treatment sections in positive (ESI +) and negative (ESI-) electrospray mode — terminology adopted from Bader et al. (2017)

log2FC level	Compound’s change	Numbers
infinity	Eliminated	3145
minus infinity	Formed	3352
between − 1 − + 1	Consistent	1225
log2FC ≥ 1	Decreased	363
log2FC ≤ − 1	Increased	408

Sensitive NT analysis can detect more signals than is possible with target methods (Bader et al., 2017). However, not all newly formed or consistent compounds can potentially be harmful to organisms and human health. Further prioritization and identification are needed to evaluate the potential risk. NTS could detect compounds with carcinogenic effects (Diana et al., 2019; Kali et al., 2021). However, some already known chlorination byproducts cannot be principally detected by the proposed method, e.g. trihalomethanes which belong to volatile compounds.

#### Treatment effect assessment for technology steps

We replaced removal efficiency with calculating treatment effects for corresponding treatment steps using the same formula and subtracting formed compounds (having log2FC level − Infinity) (results in Table 2). DWTP A showed the best treatment effect in autumn when ozonation was on considering compound number. The high efficiency of treatment effect in section Inlet/before GAC means that the advanced oxygenation step is crucial for the treatment. However, at the same time, ozonation led to a high number of formed compounds in the consequent section. These results contrast with Bader et al. (2017), which reported that the ozonation step formed only 5% of compounds present after this step. However, Bader’s study did not follow the rest of the treatment. We detected 567 formed compounds from 958 compounds detected in the sampling point after ozonation and 670 formed compounds in the following section. Our case study results report relevant compounds formed not only in section Inlet/before GAC where ozonation was applied but also in the section before GAC/Outflow. There is a presumption that ozone transformation processes can continue through the subsequent treatment. Spring results from DWTP A confirmed the downgrading of treatment effect without ozonation, a lower amount of eliminated and decreased compounds, and a lower amount of formed and increased ones.

**Table 2:** Calculated treatment effects for DWTPs divided into treatment sections based on compound numbers and areas

		Treatment effect — number			Treatment effect — area		
	Treatment section	I/O	I/b GAC	b GAC/O	I/O	I/b GAC	b GAC/O
Season	Location						
Spring	DWTP A	53%	54%	59%	25%	22%	37%
Autumn	DWTP A	73%	72%	45%	30%	19%	52%
Spring	DWTP B	37%	65%	20%	24%	40%	21%
Autumn	DWTP B	61%	46%	56%	27%	23%	25%

The overall treatment effect for DWTP B calculated from compound numbers shows a slight regression in spring for an unknown reason. We can only deduce a lower GAC filtration section effectivity (I/b GAC had a high removal effect). Treatment effects calculated from compound areas principally supported number-based trends, with one exception. DWTP A on autumn in section I/b GAC showed a much lower removal when area data instead of numbers were used. However, compound areas as signal intensity from NT analysis do not strictly correspond to quantity because various matrix or ionization effects described by Kruve, 2019 and concentration cannot be quantitatively described. Because of this reason, a more conservative treatment evaluation seems to be compound numbers. However, peak areas are potentially helpful for sample comparison without any concentration consideration.

#### Compounds with a halogenated isotope pattern

NT screening does not differ between threatening micropollutants and harmless natural compounds and detects a high number of signals. Advanced compound filtration and prioritization based on isotope patterns support identification and risk assessment (Zahn & Frömel, 2020). Compounds of interest like organohalogen compounds can be identified by the isotopic pattern of chlorine (Cl) and bromine (Br). These MPs are considered to originate from anthropogenic activities to be xenobiotic and potentially toxic. The number of chlorinated and brominated compounds can bring a closer view to the treatment efficiency of these xenobiotics and their potential formation during the ozonation or chlorination. Chlorine addition for disinfection is the last treatment step and can vary in used chlorination agents. Therefore, proportions between inlet, prior GAC, and outlet samples were examined to discover treatment-born compounds and their number. The workflow filtered compounds in two ways, one for all Cl, Br, and combination patterns and one for Cl patterns only, both in positive and negative ionization modes. The search results are presented in Table 3, containing numbers and total areas of compounds with the identified pattern.

**Table 3:** Compounds with an isotope pattern of chlorine or bromine (and combination) and only of chlorine. DWTP A has an inlet from the river, and DWTP B has an inlet from the water reservoir

			Cl + Br	Cl + Br	Cl	Cl
Season	Location	Sampling sites	Number	Sum of areas	Number	Sum of areas
Spring	DWTP A	Inlet	138	4.4.10^8^	134	4.3.10^8^
		Before GAC	128	5.4.10^8^	124	5.2.10^8^
		Outlet	194	4.7.10^8^	191	4.7.10^8^
Autumn	DWTP A	Inlet	182	7.5.10^8^	171	7.3.10^8^
		Before GAC	104	3.2.10^8^	103	3.2.10^8^
		Outlet	173	6.2.10^8^	171	6.2.10^8^
Spring	DWTP B	Inlet	119	6.2.10^8^	93	2.5.10^8^
		Before GAC	152	7.5.10^8^	149	6.3.10^8^
		Outlet	107	5.4.10^8^	93	3.1.10^8^
Autumn	DWTP B	Inlet	84	2.7.10^8^	82	2.7.10^8^
		Before GAC	77	2.3.10^8^	75	2.3.10^8^
		Outlet	61	1.5.10^8^	61	1.5.10^8^

Chlorinated compounds prevail over the bromine patterns in all samples significantly. The number and peak areas are higher for river water than for the reservoir. However, the season affected the sources differently. The river showed a higher number and total peak area of halogenated compounds in the autumn, but the reservoir did in spring. There is also an apparent difference in the number of compounds during treatment steps after GAC. While treatment-born halogenated chemicals are formed in DWTP A, the number of compounds along the technology line in DWTP B has descending trend.

The same procedure for all compounds was used to divide the compounds into levels by log2FC value. Consequently, compounds formed in the treatment section were subtracted (SM1 Table S8), and the treatment effect was calculated. The comparison of the removal is presented in Table 4. DWTP A shows the significant formation of halogenated compounds in autumn sampling. However, the increase in peak area responsible for negative removal is related to the number of compounds with highly diverse MW and retention time, as seen in Fig. 1. We could match some of these compounds with libraries available in the software. The identity of compounds, e.g. dichlorobenzoic acid or chlorosalicyl acid, must be confirmed with an injection of the standards because the number of possible isomers to these compounds can occur in the samples. 5-Chlorosalicyl acid appeared on the carcinogenic disinfection byproducts list (Kali et al., 2021). That procedure is optional if we need to continue research in the direction of targeted analysis. Anyhow, the data set acquired with the proposed method can be exploited anytime for such purposes.

**Table 4:** Calculated treatment effects for DWTPs divided into treatment sections based on the numbers and areas of compounds with a Cl and Br isotopic pattern. Negative values mean greater total area after treatment than before it. *Section I/O*, inlet/outlet; *I/b GAC*, inlet/before GAC; *b GAC/O*, before GAC/outlet

		Treatment effect — number			Treatment effect — area		
	Treatment section	I/O	I/b GAC	b GAC/O	I/O	I/b GAC	b GAC/O
Season	Location						
Spring	DWTP A	38%	46%	48%	33%	0%	51%
Autumn	DWTP A	69%	66%	36%	40%	67%	− 45%
Spring	DWTP B	55%	52%	57%	71%	49%	36%
Autumn	DWTP B	64%	30%	57%	52%	17%	43%

### Formed compounds

**Fig. 6:**
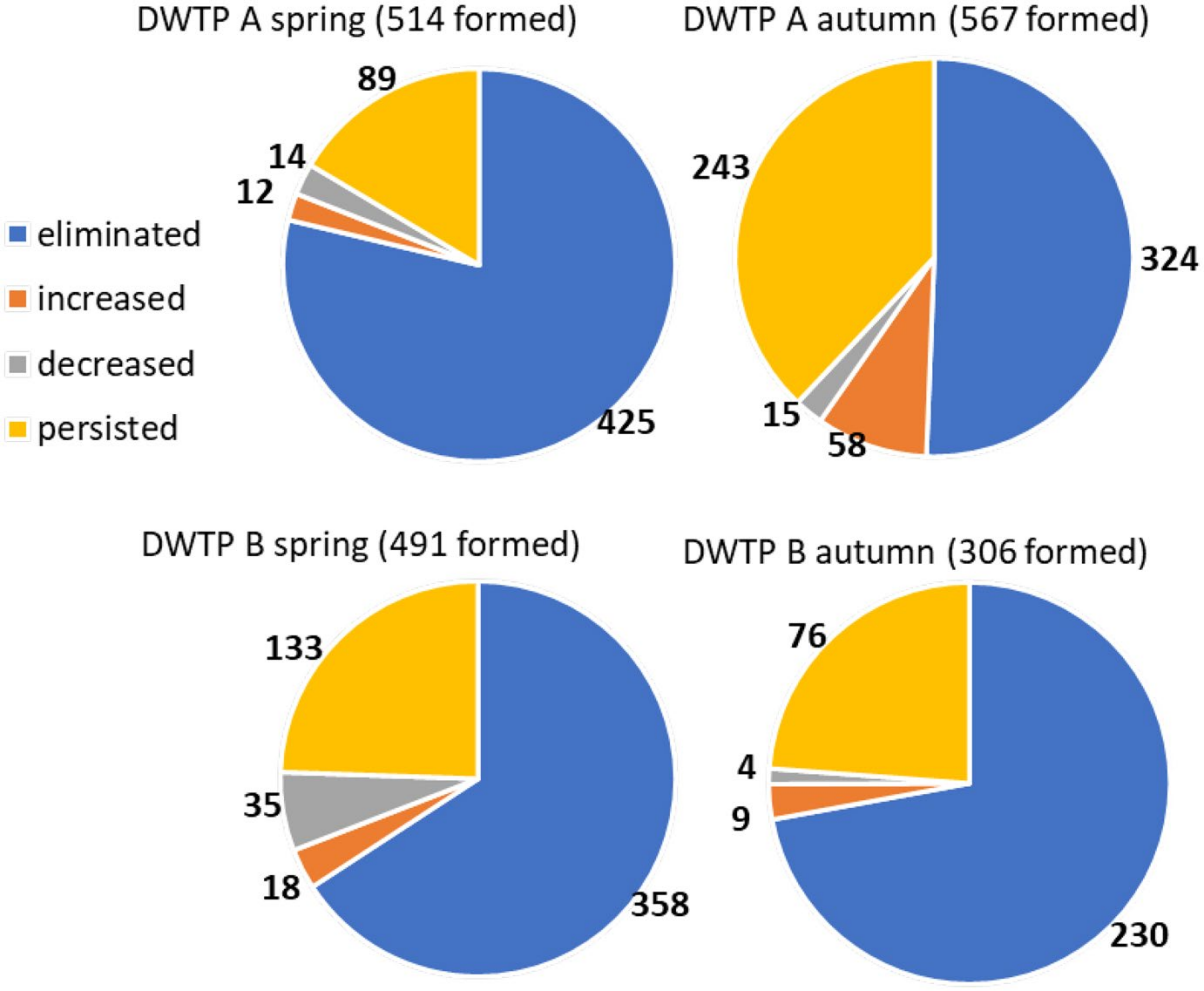
Fate of compounds formed prior to GAC in consequent treatment

Products or new chemicals formed through drinking water treatment can become part of the evaluation. Itzel (2020) studied the ozonation effect in wastewater treatment. He tracked 486 peaks eliminated by ozone and 241 peaks formed. After biological post-treatment, he found only 11 residual compounds. Due to individual detected compound levels sorting, we could track formed compounds (especially by ozonation) fate further throw treatment and divide them again by log2FC values (Fig. 6). Approximately three-quarters of compounds formed in the first treatment section were eliminated, and one-third persisted through the following treatment section, except for DWTP A in autumn when the ozonation step caused the main variance. Only half of the formed compounds were eliminated, and 43% persisted in the final drinking water. Our case study confirmed the results of the previous experiment (Von Gunten, 2003), mentioning persistent ozonation transformation products.

## Conclusion

This case study showed that applying nontarget analysis without time-consuming identifying compounds can be essential for water treatment control. Explorative analysis of targeted and nontargeted data showed a similar primary picture. The evaluation of the raw water used as a source showed the relation between season, water flow, and micropollutant content. However, assessing the treatment efficiency from NTS detected compounds in samples is crucial for understanding the treatment process as complex. Broad-range multiresidual analyses have not revealed the formation of the treatment byproducts.

Sampling points placed at influents and effluents and between treatment steps provided a unique overview of compounds change after GAC filters or ozonation. Evaluation of the treatment efficiency based only on detected compounds numbers and areas has several issues like inconsistency in peak detection, ion signals intensity affected by matrix, and recently unconventional and software-limited data processing. Nevertheless, this study on real DWTPs showed the capability of a nontarget approach combined with passive sampling. Our approach lies in an innovative combination of three main parts (real samples from different treatment stages, target and nontarget analysis, and formed compounds selection) and results evaluation based on their mutual interaction. We were able to trace formed compounds and localize their main source processes. We confirmed that ozonation compensated more pollutant-loaded raw water and removed most micropollutants.

On the other hand, most transformation products are formed during and after ozonation processes, including chlorination. In addition, we demonstrated the exploitation of data by suspect screening and identifying compounds of interest. Passive sampling connected with nontarget analysis is a promising approach for water treatment control. We believe this approach has the potential, especially for long-term monitoring of changes in technology lines, because passive sampling dramatically reduces the number of samples and provides time-weighted average information for 2 to 4 weeks. The proposed approach may help to improve treatment technologies because safe drinking water is essential in the concept of One Health.

## Supplementary Information

Below is the link to the electronic supplementary material.

Supplementary file1 (DOCX 151 KB)

Supplementary file2 (DOCX 76 KB)

## Data Availability

The data supporting this study’s findings are available from the corresponding author, Petra Nováková, upon reasonable request.

## Abbreviations

CD: Compound discoverer
DWTP: Drinking water treatment plant
ESI: Electrospray ionization
GAC: Granular active carbon
HRMS: High-resolution mass spectrometry
LC: Liquid chromatography
Log2FC: Log2Fold change
MW: Molecular weight
*m/z*: Mass/charge
MPs: Micropollutants
MS/MS: Tandem mass spectrometry
NT: Nontarget
PCA: Principal component analysis
POCIS: Polar organic chemical integrative samplers
RE: Removal efficiency
RT: Retention time
TE: Treatment effect
TPs: Transformation products
WWTP: Wastewater treatment plant
