# Supplementary material for: Novel nontarget LC-HRMS-based approaches for evaluation of drinking water treatment
Source: Environ Monit Assess. 2023 May 26;195(6):739. doi: 10.1007/s10661-023-11348-w (PMC10219882; doi:10.1007/s10661-023-11348-w)
Supplement: Supplementary file 1 — Supplementary file1 (DOCX 151 KB) [file 10661_2023_11348_MOESM1_ESM.docx]

**Novel nontarget LC-HRMS-based approaches for evaluation of drinking water treatment**

**Supplementary materials 1 – Sampling and results**

Petra Nováková^a^*, Helena Švecová^a^, Adam Bořík^a^, Roman Grabic^a^

*^a^University of South Bohemia in České Budějovice, Faculty of Fisheries and Protection of Waters, South Bohemian Research Centre of Aquaculture and Biodiversity of Hydrocenoses, Zátiší 728/II, CZ-389 25, Vodňany, Czech Republic*

* Corresponding author. Tel.: +420 38777 4752; E-mail address: novakovapetra@frov.jcu.cz; Faculty of Fisheries and Protection of Waters, Zátiší 728/II, 389 25 Vodňany, Czech Republic

|  | **keywords: nontarget/non-target; efficiency; evaluation; fold change; drinking; water** |  |  |  |
| --- | --- | --- | --- | --- |
|  | DW - drinking water, WW - waste water |  |  |  |
| **Reference** | **Description** | **MS analyser** | **Software tool** | **Sample type** |
| Nürenberg et al. 2015 | WW; post-processing and reduction of false positive results, efficiency of biological treatment step and compounds formation based on numbers | QTOF | MarkerView + MultiQuant | water |
| Parry and Young 201 | pilot experiment efficiency of advanced oxidation process (UV/H2O2), comparison of target and nontarget with removal efficiency of known analytes | QTOF | MassHunter | water |
| Itzel et al. 2020 | WW, peaks number formed by ozonation and calculation of reduced compounds | Orbitrap Q-Exactive | MZmine + R, | water |
| Bader et al. 2017 | DW; method validation, efficiency of ozonation according to fold change of peaks hight | TripleTOF | MarkerView + MultiQuant | water |
| Müller et al. 2011 | DW + surface water; detecting of all compounds and comparing them during treatment | QTOF | MassHunter + MFG, MFE | water |
| Nürenberg et al. 2019 | WW; target and nontarget removal efficiency from concentration and peak areas | Q-TOF | MarkerView + MultiQuant | water |
| Bader et al. 2016 | non-target data evaluation, method validation for repeatability increasing | Q-TOF | MarketView + MultiQuant | water |
| Schollée et al. 2016 | review of statistical approaches for processing non-target data and transformation products - log2FoldChange |  | R | water |
| Tuerk, 2016 (ACS) | WW; ozonation driven formed compounds characterisation based on ratios | Q-Exactive | Compound Discoverer | water |
| Reymond et al. 2022 | WW; fold-change between features in samples | (LTQ-FT) Orbitrap mass | Compound Discoverer | water |

SM1 Table S1. Literature review done by search in Web of Science

**References**

Bader T, Schulz W, Kümmerer K, Winzenbacher R (2017) LC-HRMS Data Processing Strategy for Reliable Sample Comparison Exemplified by the Assessment of Water Treatment Processes. Anal Chem 89:13219–13226. https://doi.org/10.1021/acs.analchem.7b03037

Bader T, Schulz W, Lucke T (2016) Application of non-target analysis with LC-HRMS for the monitoring of raw and potable water: Strategy and results. In: ACS Symposium Series. American Chemical Society, pp 49–70

Itzel F, Baetz N, Hohrenk LL, et al (2020) Evaluation of a biological post-treatment after full-scale ozonation at a municipal wastewater treatment plant. Water Res 170:115316. https://doi.org/10.1016/j.watres.2019.115316

Müller A, Schulz W, Ruck WKL, Weber WH (2011) A new approach to data evaluation in the non-target screening of organic trace substances in water analysis. Chemosphere 85:1211–1219. https://doi.org/10.1016/j.chemosphere.2011.07.009

Nürenberg G, Kunkel U, Wick A, et al (2019) Nontarget analysis: A new tool for the evaluation of wastewater processes. Water Res 163:114842. https://doi.org/10.1016/j.watres.2019.07.009

Nürenberg G, Schulz M, Kunkel U, Ternes TA (2015) Development and validation of a generic nontarget method based on liquid chromatography - high resolution mass spectrometry analysis for the evaluation of different wastewater treatment options. J Chromatogr A 1426:77–90. https://doi.org/10.1016/j.chroma.2015.11.014

Parry E, Young TM (2016) Comparing targeted and non-targeted high-resolution mass spectrometric approaches for assessing advanced oxidation reactor performance. Water Res 104:72–81. https://doi.org/10.1016/j.watres.2016.07.056

Reymond N, Emke E, Boucheron T, et al (2022) Retrospective suspect and non-target screening combined with similarity measures to prioritize MDMA and amphetamine synthesis markers in wastewater. Sci Total Environ 811:152139. https://doi.org/10.1016/j.scitotenv.2021.152139

Schollée JE, Schymanski EL, Hollender J (2016) Statistical Approaches for LC-HRMS Data to Characterize, Prioritize, and Identify Transformation Products from Water Treatment Processes. ACS Symp Ser 1241:45–65. https://doi.org/10.1021/BK-2016-1241.CH004

SM1 Table S2. Description of drinking water treatment plants.

|  | **DWTP A** | **DWTP B** |
| --- | --- | --- |
| Water source | River | Water reservoir |
| Connected consumers | 210 000 | 400 000 |
| Capacity (l/s) | 1 000 | 1 450 |
| Water production per day (m^3^) | 37 000 | 43 200 |
| Treatment process steps | Raw water collection  Coagulation + Sedimentation  Rapid sand filtration  Ozonation  GAC  Hardening  UV + Cl_2_ chlorination  Accumulation in tank | Raw water collection  Coagulation + Flotation  Rapid sand filtration  GAC  NaClO chlorination  Accumulation in tank |

SM1 Table S3. Sampling sites and information about sampling time and raw water flow

^1^ Sampling crushed by accident, inapplicable samples, ^2^ Repeated sampling

|  | **Sampling locality** | **Sampling site** | **Employment date** | **Time** | **Temperature, °C** | **Deployment date** | **Time** | **Temperature, °C** | **Flow, m^3^** |
| --- | --- | --- | --- | --- | --- | --- | --- | --- | --- |
| **Spring** | **DWTP A** | **Inlet** | 4/5/2019 | 10:15 | 10.5 | 4/18/2019 | 9:30 | 9.8 | 4.319 |
|  |  | **before GAC** | 4/5/2019 | 10:15 | 11.0 | 4/18/2019 | 9:45 | 6.8 |  |
|  |  | **Outlet** | 4/5/2019 | 10:30 | 11.3 | 4/18/2019 | 9:55 | 7 |  |
|  | **DWTP B** | **Inlet** | 4/25/2019 | 9:45 | 5.5 | 5/9/2019 | 10:00 | 9.8 | 2.542 |
|  |  | **before GAC** | 4/25/2019 | 10:00 | 5.0 | 5/9/2019 | 10:15 | 10 |  |
|  |  | **Outlet** | 4/25/2019 | 10:05 | 5.7 | 5/9/2019 | 10:20 | 10 |  |
|  |  |  |  |  |  |  |  |  |  |
| **Autumn** | **DWTP A^2^** | **Inlet** | 10/7/2019 | 10:40 | 10.5 | 10/21/2019 | 11:00 | 12 | 2.724 |
|  |  | **before GAC** | 10/7/2019 | 11:00 | 11.5 | 10/21/2019 | 11:30 | 12.5 |  |
|  |  | **Outlet** | 10/7/2019 | 11:15 | 12.0 | 10/21/2019 | 10:55 | 13 |  |
|  | **DWTP A^3^** | **Inlet** | 10/21/2019 | 11:00 | 12.0 | 11/4/2019 | 10:55 | 9 | 1.237 |
|  |  | **before GAC** | 10/21/2019 | 11:30 | 12.5 | 11/4/2019 | 11:10 | 8.5 |  |
|  |  | **Outlet** | 10/21/2019 | 10:55 | 13.0 | 11/4/2019 | 10:45 | 9 |  |
|  | **DWTP B** | **Inlet** | 10/2/2019 | 10:30 | 7.0 | 10/16/2019 | 10:30 | 7 | 0.881 |
|  |  | **before GAC** | 10/2/2019 | 10:45 | 7.0 | 10/16/2019 | 10:30 | 7.5 |  |
|  |  | **Outlet** | 10/2/2019 | 11:00 | 6.5 | 10/16/2019 | 10:45 | 7 |  |

SM1 Table S4. Target analytes concentrations (ng/POCIS) and limits of quantification (LOQ). ^1^ Type 1 are benzotriazoles, type 2 pesticides and Type 3 are pharmaceuticals.

|  |  | DWTP A spring | | | DWTP A autumn | | | DWTP B spring | | | DWTP B autumn | | |  |
| --- | --- | --- | --- | --- | --- | --- | --- | --- | --- | --- | --- | --- | --- | --- |
| Type^1^ | Analytes name | Inlet | before GAC | Outlet | Inlet | before GAC | Outlet | Inlet | before GAC | Outlet | Inlet | before GAC | Outlet | LOQ |
| 1 | 1H-benzotriazol | 14 | 61 | 2.1 | 99 | 4.5 | <0.19 | 11 | 14 | 1.7 | 12 | 7.6 | <0.19 | 0.15-0.27 |
| 1 | 1H-benzotriazol_(5/4)-methyl | 6 | 29 | <0.13 | 64 | <0.029 | <0.023 | 4 | 4.3 | <0.13 | 4.4 | 1.8 | 0.051 | 0.023-0.047 |
| 1 | 1H-benzotriazol_1-methyl | <1.3 | <1.1 | <0.95 | <1 | 2.1 | <0.56 | <1.5 | <1.2 | <0.98 | <1 | <0.84 | <1.1 | 0.56-1.1 |
| 2 | 2.4.5-trichlorophenoxyacetic_acid | <0.58 | <0.54 | <0.64 | <1.4 | <0.96 | <0.73 | <0.58 | <0.61 | <0.56 | <1.9 | <1.4 | <1.4 | 0.73-1.9 |
| 2 | 2.4-D | <0.81 | <0.72 | <0.69 | <2.7 | <1.9 | <1.4 | 1.6 | 3.1 | <0.62 | <3.4 | <2.4 | <2.5 | 1.4-3.4 |
| 2 | 2.4-Dichlorphenoxypropionic_acid | <0.46 | <0.42 | <0.5 | <1.1 | <0.72 | <0.55 | <0.46 | <0.48 | <0.44 | <1.5 | <1 | <1 | 0.55-1.5 |
| 2 | 3-chloro-4-methylaniline | <0.088 | <0.076 | <0.064 | <0.27 | <0.19 | <0.15 | <0.098 | <0.082 | <0.065 | <0.27 | <0.22 | <0.3 | 0.15-0.3 |
| 2 | 4-Isopropylaniline | <0.44 | <0.38 | <0.32 | <1.3 | 3.8 | <0.78 | <0.49 | <0.41 | <0.32 | <1.3 | <1.2 | <1.6 | 0.78-1.6 |
| 2 | Acetochlor | <0.16 | <0.14 | <0.21 | <0.11 | <0.1 | <0.093 | <0.15 | <0.14 | <0.14 | <0.17 | <0.11 | <0.16 | 0.093-0.17 |
| 2 | Acetochlor_ESA | 15 | 41 | 15 | 29 | <0.61 | 0.64 | 22 | 30 | 18 | 29 | 28 | 17 | 0.61-1.6 |
| 2 | Acetochlor_OA | <0.39 | 0.61 | <0.35 | <2.3 | <1 | <1.1 | <0.42 | <0.48 | <0.43 | <3.3 | <2.6 | <1.9 | 1-3.3 |
| 2 | Alachlor | <0.16 | 0.21 | <0.21 | <0.089 | <0.077 | <0.071 | <0.15 | 0.2 | <0.13 | <0.13 | <0.083 | <0.12 | 0.071-0.13 |
| 2 | Alachlor_ESA | 18 | 55 | 19 | 94 | <10 | <10 | 31 | 46 | 29 | 86 | 37 | 31 | 10-26 |
| 2 | Alachlor_OA | 0.33 | 0.53 | <0.25 | <1.5 | <0.85 | <0.88 | 0.52 | 0.74 | 0.51 | <2.2 | <2.2 | <1.6 | 0.85-2.2 |
| 2 | Ametryn | <0.26 | <0.23 | <0.22 | <0.24 | <0.18 | <0.13 | <0.26 | <0.26 | <0.2 | <0.3 | <0.24 | <0.25 | 0.13-0.3 |
| 2 | Anthranilic_acid_isopropylamide | <0.031 | 0.038 | <0.026 | <0.16 | 0.12 | <0.1 | <0.039 | <0.03 | <0.027 | <0.16 | <0.11 | <0.19 | 0.1-0.19 |
| 2 | Atraton | <0.22 | <0.19 | <0.16 | <0.16 | 0.13 | <0.086 | <0.25 | <0.2 | <0.16 | <0.16 | <0.13 | <0.18 | 0.086-0.18 |
| 2 | Atrazine | 3.6 | 14 | 1.1 | 5.3 | 0.72 | 0.12 | 1.7 | 4 | 0.4 | 1.6 | 0.81 | 0.36 | 0.12-0.26 |
| 2 | Atrazine_2-hydroxy | 11 | 30 | 3.8 | 5.3 | 1.4 | 0.32 | 14 | 14 | 2.8 | 1.7 | 1.4 | 0.44 | 0.085-0.15 |
| 2 | Atrazine_desethyl | 3.1 | 9.7 | 1.3 | 9.1 | 5.2 | 0.65 | 2.3 | 3.2 | 0.77 | 3.7 | 2.1 | 1.2 | 0.09-0.18 |
| 2 | Atrazine_desethyl-2-hydroxy | 0.26 | 0.31 | 0.22 | <0.26 | <0.19 | <0.17 | 0.42 | 0.3 | 0.26 | <0.25 | <0.18 | <0.31 | 0.89-1.6 |
| 2 | Atrazine_desisopropyl | 0.34 | 1.1 | 0.23 | 0.95 | 0.71 | <0.074 | 0.32 | 0.72 | <0.2 | 0.28 | 0.53 | <0.13 | 0.074-0.13 |
| 2 | Azoxystrobin | <0.16 | <0.13 | <0.22 | 0.16 | <0.085 | <0.086 | <0.14 | <0.14 | <0.13 | <0.22 | <0.087 | <0.15 | 0.085-0.22 |
| 2 | Bensulfuron_methyl | <0.39 | <0.34 | <0.33 | <0.21 | <0.2 | <0.14 | <0.39 | <0.39 | <0.3 | <0.26 | <0.25 | <0.27 | 0.14-0.27 |
| 2 | Bentazone | 1.4 | 4.6 | <0.24 | 4.3 | <0.13 | <0.13 | 0.36 | 0.75 | <0.3 | 0.82 | 0.51 | <0.25 | 0.13-0.37 |
| 2 | Carbendiazim | <0.23 | 0.24 | <0.2 | 0.32 | <0.15 | <0.13 | <0.29 | <0.23 | <0.2 | <0.19 | <0.14 | <0.23 | 0.13-0.23 |
| 2 | Carbofuran-3-hydroxy | 0.46 | 0.13 | <0.081 | 1.3 | 0.94 | 0.36 | 0.75 | 0.29 | 0.29 | 0.6 | <0.034 | <0.046 | 0.022-0.046 |
| 2 | Chlorantraniliprole | <0.69 | <0.61 | <0.58 | <0.15 | <0.11 | <0.082 | <0.7 | <0.69 | <0.53 | <0.18 | <0.14 | <0.15 | 0.082-0.18 |
| 2 | Chloridazon | <0.27 | <0.24 | <0.2 | <0.36 | <0.28 | <0.21 | <0.3 | <0.25 | <0.2 | <0.37 | <0.32 | <0.44 | 0.21-0.44 |
| 2 | Chloridazon_desphenyl | 4.9 | 6.2 | 3.6 | 1.8 | 0.26 | <0.22 | 8.5 | 5.8 | 6.5 | 1.4 | 0.63 | 0.59 | 0.22-0.4 |
| 2 | Chloridazon_methyl_desphenyl | 1.1 | 2.6 | <0.23 | 1.9 | <0.2 | <0.18 | 2.3 | 2.1 | 0.36 | 1.4 | 1.5 | <0.32 | 0.18-0.32 |
| 2 | Chlorotoluron | 8 | 18 | <0.61 | 3 | <0.22 | <0.16 | 3.5 | 17 | <0.55 | 3.3 | 1.5 | <0.29 | 0.16-0.36 |
| 2 | Chlorotoluron_desmethyl | <0.73 | 0.72 | <0.62 | <0.11 | <0.085 | <0.062 | <0.74 | <0.74 | <0.56 | <0.14 | <0.11 | <0.11 | 0.062-0.14 |
| 2 | Clomazone | <0.33 | <0.3 | <0.28 | 0.45 | <0.13 | <0.093 | <0.34 | <0.33 | <0.25 | <0.22 | <0.16 | <0.17 | 0.093-0.22 |
| 2 | Cyproconazole | <0.13 | 0.39 | <0.17 | 12 | 0.78 | <0.085 | <0.11 | <0.11 | <0.1 | <0.22 | <0.085 | <0.15 | 0.084-0.22 |
| 2 | DEET | 61 | 64 | 35 | 44 | 25 | 11 | 46 | 55 | 43 | 15 | <5.3 | 26 | 2.9-5.8 |
| 2 | Desmetryn | <0.26 | <0.23 | <0.22 | <0.23 | <0.18 | <0.13 | <0.26 | <0.26 | <0.2 | <0.29 | <0.23 | <0.24 | 0.13-0.29 |
| 2 | Diazinon | 0.43 | 2.7 | <0.11 | <0.13 | <0.12 | <0.11 | <0.078 | <0.068 | <0.069 | <0.2 | <0.13 | <0.2 | 0.11-0.2 |
| 2 | Dimethachlor | 0.57 | 1.6 | <0.32 | 0.95 | <0.16 | <0.12 | <0.39 | <0.38 | <0.29 | <0.26 | <0.21 | <0.22 | 0.12-0.26 |
| 2 | Dimethachlor_ESA_pos | 2.9 | 5.6 | 3.7 | 5.7 | 0.39 | 0.33 | 0.51 | 0.73 | 0.4 | 1.1 | <0.19 | 0.44 | 0.18-0.32 |
| 2 | Dimethachlor_OA | <0.21 | <0.13 | <0.17 | <0.44 | <0.3 | <0.19 | <0.18 | <0.13 | <0.15 | <0.57 | <0.34 | <0.32 | 0.19-0.57 |
| 2 | Dimethenamid_ESA | <0.49 | <0.43 | <0.36 | <0.17 | <0.12 | <0.091 | 1.2 | 1.3 | 0.72 | 0.96 | <0.14 | 0.2 | 0.091-0.19 |
| 2 | Dimethenamid_OA | <0.39 | <0.33 | <0.28 | <0.25 | <0.17 | <0.13 | <0.43 | <0.36 | <0.29 | <0.25 | <0.2 | <0.28 | 0.13-0.28 |
| 2 | Dimethoate | <0.33 | 1.1 | <0.24 | 0.66 | <0.14 | <0.1 | <0.37 | 0.49 | <0.25 | <0.19 | <0.16 | <0.21 | 0.1-0.21 |
| 2 | Dimethomorph | <0.25 | 0.29 | <0.22 | 0.43 | <0.12 | <0.089 | <0.26 | <0.26 | <0.2 | <0.16 | <0.16 | <0.16 | 0.089-0.16 |
| 2 | Diuron_desmethyl | <0.31 | <0.27 | <0.26 | <0.27 | <0.21 | <0.15 | <0.31 | <0.31 | <0.24 | <0.34 | <0.27 | <0.28 | 0.15-0.34 |
| 2 | Epoxiconazole | <0.13 | <0.11 | <0.18 | <0.11 | <0.079 | <0.08 | <0.11 | <0.11 | <0.11 | <0.16 | <0.08 | <0.14 | 0.079-0.16 |
| 2 | Fenuron | <0.3 | <0.27 | <0.26 | 0.46 | <0.23 | <0.2 | <0.39 | <0.3 | <0.27 | <0.25 | <0.21 | <0.35 | 0.2-0.35 |
| 2 | Florasulam | <0.1 | <0.089 | <0.082 | <0.26 | <0.18 | <0.14 | <0.094 | <0.092 | <0.1 | <0.33 | <0.24 | <0.25 | 0.14-0.33 |
| 2 | Fluazifop-p | <0.2 | <0.16 | <0.27 | 1.7 | <0.09 | <0.091 | <0.17 | <0.17 | <0.16 | <0.23 | <0.091 | <0.16 | 0.09-0.23 |
| 2 | Flusilazole | <0.15 | <0.13 | <0.19 | <0.12 | <0.1 | <0.094 | <0.14 | <0.12 | <0.12 | <0.18 | <0.11 | <0.16 | 0.094-0.18 |
| 2 | Hexazinone | 1.5 | 4 | 1.3 | 2.3 | <0.26 | 0.26 | 1.7 | 2.2 | 1.2 | 2.7 | 1.7 | 1.5 | 0.19-0.44 |
| 2 | Imazamethabenz_methyl | <0.28 | <0.25 | <0.23 | <0.3 | <0.21 | <0.16 | <0.28 | <0.28 | <0.21 | <0.38 | <0.27 | <0.29 | 0.16-0.38 |
| 2 | Imazamox | <0.083 | <0.072 | <0.07 | 0.092 | <0.042 | <0.036 | <0.1 | <0.081 | <0.071 | 0.69 | 0.19 | <0.065 | 0.036-0.065 |
| 2 | Imidacloprid | 3.9 | 21 | <0.17 | 2.6 | <0.098 | <0.084 | 0.28 | <0.2 | <0.17 | 0.19 | 0.16 | <0.15 | 0.084-0.15 |
| 2 | Isoproturon | 0.28 | 1 | <0.21 | 0.51 | <0.2 | <0.15 | <0.24 | <0.23 | <0.25 | <0.24 | <0.32 | <0.27 | 0.15-0.32 |
| 2 | Isoproturon_monodemethyl | <0.45 | <0.41 | <0.39 | <0.26 | 0.44 | <0.16 | <0.46 | <0.46 | <0.35 | <0.33 | <0.28 | <0.29 | 0.16-0.33 |
| 2 | Lenacil | <0.71 | <0.63 | <0.6 | 0.12 | 1.9 | 0.61 | <0.72 | <0.72 | <0.55 | <0.077 | <0.059 | <0.062 | 0.033-0.077 |
| 2 | Linuron | <0.26 | <0.21 | <0.36 | <0.17 | <0.12 | <0.12 | <0.23 | <0.22 | <0.22 | <0.24 | <0.12 | <0.21 | 0.12-0.24 |
| 2 | Malathion | <1.6 | <1.3 | <2.2 | <1.3 | <0.91 | <0.92 | <1.4 | <1.3 | <1.3 | <1.9 | <0.92 | <1.6 | 0.91-1.9 |
| 2 | MCPA | 1.1 | 1.3 | <0.5 | 1.5 | <0.57 | <0.43 | <0.47 | 0.53 | <0.46 | <1.2 | <0.8 | <0.8 | 0.43-1.2 |
| 2 | MCPP | <0.48 | <0.43 | <0.51 | <0.95 | <0.78 | <0.6 | <0.48 | <0.51 | <0.47 | <1.4 | <1.1 | <1.1 | 0.6-1.4 |
| 2 | Metalaxyl | <0.2 | 0.22 | <0.16 | <0.21 | <0.16 | <0.13 | <0.18 | <0.18 | <0.2 | <0.19 | <0.27 | <0.22 | 0.13-0.27 |
| 2 | Metazachlor | 0.81 | 1.6 | 0.7 | 1.9 | <0.21 | 0.19 | 7.7 | 11 | 3.2 | 1.6 | 1.4 | 1.3 | 0.15-0.33 |
| 2 | Metazachlor_ESA | 32 | 40 | 26 | 33 | <1.6 | <1 | 29 | 31 | 27 | 58 | 28 | 19 | 1-3.9 |
| 2 | Metazachlor_OA | 9.1 | 7.4 | 4.5 | 3.3 | <0.14 | 0.23 | 3.6 | 4 | 3.2 | 5.5 | 3 | 2.4 | 0.089-0.32 |
| 2 | Metconazole | <0.12 | <0.099 | <0.16 | <0.15 | <0.082 | <0.082 | <0.11 | <0.1 | <0.1 | <0.21 | <0.083 | <0.14 | 0.082-0.21 |
| 2 | Methabenzthiazuron | <0.47 | <0.42 | <0.4 | <0.19 | <0.15 | <0.11 | <0.48 | <0.47 | <0.36 | <0.24 | <0.19 | <0.2 | 0.11-0.24 |
| 2 | Methoxyfenozide_pos | 0.21 | 0.26 | <0.15 | <0.16 | <0.11 | <0.11 | <0.096 | <0.093 | <0.092 | <0.23 | <0.11 | <0.2 | 0.11-0.23 |
| 2 | Metobromuron | <0.5 | <0.44 | <0.42 | <0.18 | <0.13 | <0.098 | <0.51 | <0.5 | <0.38 | <0.23 | <0.17 | <0.18 | 0.098-0.23 |
| 2 | Metolachlor | 0.29 | 0.44 | <0.27 | 0.29 | <0.099 | <0.1 | 0.28 | 0.29 | 0.17 | 0.47 | 0.33 | <0.17 | 0.099-0.26 |
| 2 | Metolachlor_ESA | 15 | 25 | 11 | 6.7 | 0.59 | 0.73 | 15 | 15 | 7.7 | 7.4 | 2.8 | 4.5 | 0.16-0.28 |
| 2 | Metolachlor_OA | <1.1 | <0.98 | <0.93 | <0.32 | <0.24 | <0.18 | <1.1 | <1.1 | <0.84 | <0.39 | <0.31 | <0.33 | 0.18-0.39 |
| 2 | Metoxuron | <0.32 | <0.29 | <0.28 | <0.47 | <0.35 | <0.26 | <0.33 | <0.33 | <0.25 | <0.59 | <0.46 | <0.48 | 0.26-0.59 |
| 2 | Metribuzin_desamino | <0.35 | 0.4 | <0.29 | 0.51 | <0.2 | <0.14 | <0.35 | <0.35 | <0.27 | <0.28 | <0.25 | <0.26 | 0.14-0.28 |
| 2 | Metsulfuron_methyl | <0.37 | <0.33 | <0.31 | <0.18 | <0.17 | <0.13 | <0.37 | <0.37 | <0.28 | <0.23 | <0.22 | <0.23 | 0.13-0.23 |
| 2 | Miconazole | <0.13 | <0.14 | <0.11 | <0.068 | <0.092 | <0.06 | <0.13 | <0.15 | <0.13 | <0.072 | <0.081 | <0.1 | 0.06-0.18 |
| 2 | Monolinuron | <0.49 | 0.58 | <0.42 | <0.084 | <0.061 | 0.052 | <0.5 | <0.5 | <0.38 | <0.11 | <0.079 | <0.082 | 0.045-0.11 |
| 2 | N-chloroacetyl-2.6-diethylaniline | <0.47 | <0.42 | <0.4 | <0.15 | <0.11 | <0.081 | <0.48 | <0.48 | <0.36 | <0.19 | <0.14 | <0.15 | 0.081-0.19 |
| 2 | Pirimicarb | <0.21 | 0.21 | <0.18 | 0.18 | <0.17 | <0.14 | <0.27 | <0.21 | <0.18 | <0.16 | <0.16 | <0.26 | 0.14-0.26 |
| 2 | Pirimiphos_ethyl | <0.15 | <0.12 | <0.2 | <0.13 | <0.099 | <0.1 | <0.13 | <0.13 | <0.12 | <0.19 | <0.1 | <0.17 | 0.099-0.19 |
| 2 | Pirimiphos_methyl | <0.15 | <0.12 | <0.2 | <0.15 | <0.081 | <0.082 | <0.13 | <0.12 | <0.12 | <0.21 | <0.083 | <0.14 | 0.081-0.21 |
| 2 | Prometryn | <0.16 | <0.15 | <0.14 | <0.14 | <0.12 | <0.093 | <0.15 | <0.15 | <0.17 | <0.13 | <0.2 | <0.16 | 0.093-0.2 |
| 2 | Propachlor | <0.45 | <0.4 | <0.38 | <0.11 | 0.092 | <0.057 | <0.45 | <0.45 | <0.34 | <0.13 | <0.1 | <0.1 | 0.057-0.13 |
| 2 | Propazine | <0.42 | 1.1 | <0.36 | 0.41 | <0.16 | <0.12 | <0.42 | <0.42 | <0.32 | <0.2 | <0.21 | <0.21 | 0.12-0.21 |
| 2 | Propazine_hydroxy | 0.67 | 1.7 | 0.32 | 1.3 | 0.45 | 0.12 | 1 | 0.95 | 0.24 | 0.77 | 0.54 | 0.34 | 0.12-0.21 |
| 2 | Propiconazole | 0.38 | 0.54 | <0.19 | 35 | 0.93 | 0.11 | 0.24 | 0.81 | 0.19 | <0.17 | 0.11 | <0.14 | 0.082-0.17 |
| 2 | Pyrimethanil | <0.28 | <0.25 | <0.23 | <0.28 | <0.2 | 0.18 | <0.28 | <0.28 | <0.21 | <0.35 | <0.26 | <0.27 | 0.15-0.35 |
| 2 | Sebuthylazine | <0.4 | <0.35 | <0.34 | <0.16 | <0.16 | 0.53 | <0.4 | <0.4 | <0.31 | <0.2 | <0.2 | <0.21 | 0.12-0.21 |
| 2 | Simazine | 0.23 | 0.49 | <0.2 | 0.34 | <0.12 | <0.091 | 0.33 | 0.74 | <0.18 | 0.33 | 0.23 | <0.17 | 0.091-0.17 |
| 2 | Simazine_hydroxy | <0.18 | 0.19 | <0.15 | 0.34 | <0.19 | <0.16 | <0.23 | <0.18 | <0.15 | <0.19 | <0.18 | <0.29 | 0.16-0.29 |
| 2 | Tebuconazole | 0.64 | 1.1 | <0.31 | 0.99 | 0.1 | <0.072 | 0.33 | <0.19 | <0.19 | <0.18 | <0.072 | <0.13 | 0.071-0.18 |
| 2 | Terbuthylazine | 0.52 | 0.91 | 0.62 | 1.2 | 0.22 | 0.35 | 0.56 | 0.64 | 0.34 | 0.58 | 0.6 | 0.26 | 0.13-0.3 |
| 2 | Terbuthylazine_desethyl | 2.3 | 4.2 | 4.3 | 1.9 | 1.6 | 1.1 | 2.2 | 2.9 | 1 | 1 | 0.65 | 0.55 | 0.11-0.2 |
| 2 | Terbuthylazine_desethyl-2-hydroxy | 0.88 | 1.3 | 1 | 1.1 | 2.5 | 0.71 | 0.97 | 1.4 | 0.4 | 0.73 | 0.68 | 0.34 | 0.12-0.21 |
| 2 | Terbuthylazine_hydroxy | 2.9 | 7.4 | 1 | 12 | 1.9 | 0.6 | 3.1 | 3 | 0.63 | 3.4 | 1.9 | 0.53 | 0.12-0.21 |
| 2 | Terbutryn | 0.16 | 0.41 | <0.11 | 0.95 | <0.13 | <0.097 | 0.13 | 0.17 | <0.14 | 0.17 | 0.29 | <0.17 | 0.097-0.21 |
| 2 | Thiamethoxam | <0.26 | 0.38 | <0.22 | 0.84 | 0.1 | <0.083 | <0.32 | <0.25 | <0.22 | <0.12 | <0.091 | <0.15 | 0.083-0.15 |
| 2 | Triadimenol | <0.32 | <0.28 | <0.36 | <2.2 | <1.4 | <1.2 | <0.29 | <0.29 | <0.28 | <3.4 | <1.5 | <1.9 | 1.2-3.4 |
| 2 | Triallat | <1.8 | <1.5 | <2.5 | <1.2 | <0.66 | <0.67 | <1.6 | <1.5 | <1.5 | <1.7 | <0.67 | <1.2 | 0.66-1.7 |
| 2 | Triticonazole | <0.19 | <0.16 | <0.21 | 0.61 | <0.089 | <0.072 | <0.17 | <0.17 | <0.16 | <0.22 | <0.096 | <0.12 | 0.072-0.22 |
| 3 | 10,11-dihydrocarbamazepine | <0.19 | <0.2 | <0.16 | <0.14 | <0.092 | <0.065 | <0.19 | <0.21 | <0.18 | <0.15 | <0.1 | <0.12 | 0.065-0.27 |
| 3 | 10,11-trans-dixydroxy 10,11-dihydroCBZ | 7.5 | 29 | 3.2 | 30 | 1.6 | <0.44 | 5.1 | 6.1 | 2.3 | 3.9 | 4.2 | 2.3 | 0.081-0.83 |
| 3 | Alfuzosin | <0.22 | <0.2 | <0.18 | <0.2 | <0.15 | <0.11 | <0.16 | <0.17 | <0.18 | <0.19 | <0.13 | <0.17 | 0.11-0.29 |
| 3 | Alprazolam | <0.15 | 0.5 | 0.25 | 0.19 | <0.093 | <0.066 | <0.15 | <0.17 | 0.24 | <0.12 | 0.19 | <0.13 | 0.066-0.21 |
| 3 | Amitryptyline | <0.11 | <0.11 | <0.098 | <0.12 | <0.096 | <0.063 | <0.11 | <0.099 | <0.11 | <0.12 | <0.084 | <0.11 | 0.063-0.15 |
| 3 | Atenolol | 0.27 | 0.66 | <0.093 | 1.3 | <0.2 | <0.15 | 0.98 | 1.4 | 0.13 | 0.64 | 0.57 | <0.19 | 0.081-0.2 |
| 3 | Atorvastatin | <0.14 | 0.22 | <0.11 | 0.35 | <0.056 | <0.036 | 0.14 | <0.15 | <0.13 | 0.077 | 0.096 | <0.062 | 0.036-0.19 |
| 3 | Azithromycin | <9.6 | <8.8 | <8.1 | <50 | <21 | <18 | <6.2 | <6.7 | <8 | <44 | <17 | <26 | 6.2-50 |
| 3 | Bezafibrate | <0.17 | <0.18 | <0.14 | <0.13 | <0.13 | <0.083 | <0.17 | <0.19 | 0.19 | <0.13 | <0.11 | <0.14 | 0.083-0.23 |
| 3 | Biperiden | <0.13 | <0.13 | <0.1 | <0.18 | <0.09 | <0.064 | <0.12 | <0.14 | <0.12 | <0.19 | <0.099 | <0.12 | 0.064-0.19 |
| 3 | Bisoprolol | <0.12 | 0.52 | <0.11 | 0.48 | <0.095 | <0.08 | <0.092 | <0.1 | <0.1 | <0.089 | <0.075 | <0.11 | 0.075-0.17 |
| 3 | Budenoside | <0.97 | 2.5 | <0.86 | <0.93 | <0.76 | <0.5 | <0.83 | <0.6 | <0.5 | <0.9 | 2.3 | <0.86 | 0.5-0.97 |
| 3 | Caffeine | 17 | 14 | 3.6 | 23 | 2.1 | 4.1 | 41 | 34 | 8.3 | 14 | 11 | <2 | 0.2-2 |
| 3 | Carbamazepine (CBZ) | 4.6 | 16 | 0.71 | 24 | <0.098 | 0.077 | 3 | 3.1 | 1.1 | 1.6 | 1.5 | 0.44 | 0.07-0.26 |
| 3 | Carbamazepine 10,11-epoxide | 0.72 | 2.5 | 0.25 | 2.3 | 0.2 | <0.055 | 0.39 | 0.53 | 0.35 | 0.37 | 0.31 | 0.22 | 0.055-0.25 |
| 3 | Cetirizine | 1.8 | 8.9 | <0.13 | 9.3 | <0.1 | <0.073 | 1.5 | 2 | 0.26 | 1.9 | 1.4 | <0.14 | 0.073-0.22 |
| 3 | Cilazapril | <0.11 | <0.11 | <0.09 | <0.085 | <0.1 | <0.074 | <0.11 | <0.12 | <0.1 | <0.089 | <0.11 | <0.14 | 0.074-0.15 |
| 3 | Citalopram | 0.62 | 2 | 0.14 | 1.1 | <0.13 | <0.091 | 0.19 | 0.47 | <0.14 | 0.19 | 0.2 | <0.17 | 0.091-0.21 |
| 3 | Clarithromycin | 12 | 32 | 0.42 | 16 | <2 | <1.7 | 5.9 | 9.7 | 3.3 | 7.6 | 4.5 | <2.5 | 0.24-3.3 |
| 3 | Clemastine | <0.079 | <0.082 | <0.065 | <0.069 | <0.085 | <0.056 | <0.077 | <0.087 | <0.075 | <0.072 | <0.074 | <0.095 | 0.055-0.11 |
| 3 | Clindamycin | 0.79 | 3.2 | <0.12 | 2.9 | <0.071 | <0.06 | 0.4 | 0.45 | <0.12 | 0.46 | 0.27 | <0.088 | 0.059-0.16 |
| 3 | Clindamycin_sulfoxide | 1.5 | 5.3 | <0.1 | 7.4 | <0.11 | <0.089 | 0.83 | 1.1 | 0.38 | 0.84 | 0.83 | 0.14 | 0.078-0.14 |
| 3 | Clomipramine | <0.099 | <0.1 | <0.082 | <0.067 | <0.087 | <0.057 | <0.098 | <0.11 | <0.094 | <0.07 | <0.076 | <0.098 | 0.057-0.14 |
| 3 | Clonazepam | <0.24 | <0.21 | <0.21 | <0.15 | <0.13 | <0.099 | <0.15 | <0.17 | <0.2 | <0.14 | 0.15 | <0.14 | 0.095-0.25 |
| 3 | Codeine | 0.33 | 0.86 | <0.15 | 0.35 | <0.23 | <0.17 | <0.12 | <0.12 | <0.15 | <0.21 | <0.16 | <0.22 | 0.12-0.23 |
| 3 | Diclofenac | 3.7 | 15 | 0.25 | 12 | <0.17 | <0.11 | 4.7 | 7 | 0.69 | 2.5 | 2.8 | 0.25 | 0.11-0.21 |
| 3 | Dicycloverine | <0.12 | <0.13 | <0.1 | <0.073 | <0.092 | <0.06 | <0.12 | <0.14 | <0.12 | <0.076 | <0.08 | <0.1 | 0.06-0.17 |
| 3 | Diltiazem | <0.17 | <0.18 | <0.14 | <0.15 | <0.13 | <0.09 | <0.17 | <0.19 | <0.17 | <0.16 | <0.14 | <0.17 | 0.09-0.24 |
| 3 | Diphenhydramine | <0.14 | <0.14 | <0.11 | <0.15 | <0.098 | <0.07 | <0.13 | <0.15 | <0.13 | <0.16 | <0.11 | <0.13 | 0.07-0.19 |
| 3 | Disopyramide | <0.15 | <0.14 | <0.12 | <0.16 | <0.099 | <0.074 | <0.11 | <0.12 | <0.12 | <0.16 | <0.082 | <0.16 | 0.074-0.2 |
| 3 | Donepezil | <0.12 | <0.11 | <0.096 | <0.13 | <0.14 | <0.1 | <0.086 | <0.092 | <0.097 | <0.13 | <0.11 | <0.16 | 0.072-0.16 |
| 3 | Erythromycin | 1.6 | 2.8 | <0.61 | 1.4 | <0.34 | <0.29 | 1.3 | 0.67 | <0.59 | <0.52 | <0.28 | <0.42 | 0.28-0.82 |
| 3 | Fenofibrate | <0.098 | 0.23 | <0.081 | <0.056 | <0.066 | <0.043 | 0.11 | <0.11 | 0.22 | <0.058 | <0.058 | <0.074 | 0.043-0.14 |
| 3 | Fexofenadine | 1.3 | 5.8 | 0.4 | 4.9 | <0.1 | <0.072 | 0.98 | 1.1 | 0.37 | 1.4 | 0.89 | <0.14 | 0.072-0.22 |
| 3 | Glibenclamide | <0.13 | <0.14 | <0.11 | <0.085 | <0.12 | <0.077 | <0.13 | <0.14 | <0.12 | <0.089 | <0.1 | <0.13 | 0.077-0.18 |
| 3 | Glimepiride | <0.14 | 0.24 | <0.11 | <0.11 | <0.15 | <0.096 | 0.22 | <0.15 | <0.13 | <0.11 | <0.13 | <0.16 | 0.096-0.19 |
| 3 | Haloperidol | <0.18 | <0.19 | <0.15 | <0.17 | <0.16 | <0.11 | <0.18 | <0.2 | <0.18 | <0.18 | <0.18 | <0.22 | 0.11-0.26 |
| 3 | Ioxynil | <0.2 | <0.2 | <0.18 | <0.38 | <0.19 | <0.19 | <0.22 | <0.25 | <0.22 | <0.54 | <0.48 | <0.36 | 0.19-0.54 |
| 3 | Irbesartan | 4.4 | 20 | 1.1 | 21 | 0.82 | <0.074 | 0.94 | 1.1 | 0.32 | 0.9 | 0.79 | 0.38 | 0.074-0.2 |
| 3 | Lamotrigine | 9.6 | 32 | <0.23 | 34 | 3.3 | <0.25 | 4.6 | 6 | <0.23 | 3.8 | 4.3 | <0.47 | 0.17-0.47 |
| 3 | Loperamide | <0.11 | <0.11 | <0.088 | <0.076 | <0.086 | <0.057 | <0.1 | <0.12 | <0.1 | <0.079 | <0.075 | <0.097 | 0.057-0.15 |
| 3 | Maprotiline | <0.16 | <0.16 | <0.14 | <0.095 | <0.097 | <0.063 | <0.15 | <0.14 | <0.15 | <0.099 | <0.085 | <0.11 | 0.063-0.21 |
| 3 | Meclozine | <0.11 | <0.12 | <0.095 | <0.073 | <0.093 | <0.061 | <0.11 | <0.13 | <0.11 | <0.076 | <0.082 | <0.1 | 0.061-0.16 |
| 3 | Memantine | <0.16 | 0.48 | <0.13 | 0.75 | <0.12 | <0.086 | <0.12 | <0.13 | <0.13 | <0.1 | <0.097 | <0.13 | 0.086-0.21 |
| 3 | Metamphetamine | <0.12 | <0.091 | <0.12 | 0.36 | <0.1 | <0.082 | <0.09 | <0.1 | <0.082 | <0.12 | <0.091 | <0.12 | 0.082-0.12 |
| 3 | Metoprolol | 1.6 | 7.7 | <0.11 | 9.9 | <0.14 | <0.12 | <0.093 | 0.78 | <0.11 | 0.46 | 0.26 | <0.16 | 0.093-0.2 |
| 3 | Metoprolol acid | 5.5 | 19 | 1.2 | 11 | 0.32 | <0.14 | 4.7 | 7.3 | 0.83 | 6.5 | 5.7 | 0.75 | 0.097-0.2 |
| 3 | Mianserin | <0.18 | 0.72 | <0.15 | <0.18 | <0.15 | <0.1 | <0.18 | <0.2 | <0.17 | <0.18 | <0.16 | <0.2 | 0.1-0.25 |
| 3 | Mirtazapine | 0.65 | 0.99 | <0.19 | 0.56 | <0.15 | <0.11 | <0.17 | <0.18 | <0.19 | <0.15 | <0.13 | <0.18 | 0.11-0.3 |
| 3 | N-Desmethylcitalopram | <0.17 | <0.18 | <0.14 | 0.85 | <0.12 | <0.083 | <0.17 | <0.19 | <0.16 | <0.19 | <0.13 | <0.16 | 0.083-0.24 |
| 3 | Norsertraline | <0.082 | <0.086 | <0.068 | <0.036 | <0.03 | <0.02 | <0.082 | <0.091 | <0.078 | <0.038 | <0.027 | <0.034 | 0.02-0.11 |
| 3 | O-Desmethylvenlafaxine | 8.4 | 4.8 | <0.2 | 34 | <0.22 | <0.18 | 2.8 | 2.9 | 0.25 | 3.3 | 3.6 | <0.26 | 0.15-0.29 |
| 3 | Orphenadrine | <0.12 | <0.12 | <0.096 | <0.15 | <0.099 | <0.071 | <0.11 | <0.13 | <0.11 | <0.16 | <0.11 | <0.13 | 0.071-0.16 |
| 3 | Oseltamivir carboxylate | <0.14 | <0.14 | 0.22 | <0.48 | <0.33 | <0.25 | <0.11 | <0.12 | 0.21 | <0.41 | <0.34 | <0.48 | 0.091-0.48 |
| 3 | Oxazepam | 1.5 | 3.1 | <0.14 | 2.8 | 0.34 | 2.9 | 0.18 | 0.17 | <0.13 | 0.14 | 0.15 | 0.14 | 0.063-0.17 |
| 3 | Oxcarbazepine | 0.26 | 0.22 | <0.13 | 1 | <0.11 | <0.079 | <0.15 | <0.17 | <0.15 | <0.14 | <0.12 | <0.15 | 0.079-0.22 |
| 3 | Pizotifen | <0.15 | <0.16 | <0.13 | <0.25 | <0.19 | <0.14 | <0.15 | <0.17 | <0.15 | <0.26 | <0.21 | <0.26 | 0.11-0.26 |
| 3 | Propranolol | 0.25 | <0.14 | <0.12 | 38 | <0.12 | <0.1 | <0.1 | <0.12 | <0.12 | <0.11 | <0.096 | <0.14 | 0.096-0.2 |
| 3 | Ropinirole | <0.15 | <0.14 | <0.12 | <0.12 | <0.086 | <0.073 | <0.11 | <0.12 | <0.12 | <0.11 | <0.069 | <0.1 | 0.069-0.2 |
| 3 | Rosuvastatin | 0.26 | 0.22 | <0.084 | 0.53 | <0.19 | <0.13 | 0.2 | <0.11 | <0.097 | 0.24 | 0.25 | <0.25 | 0.072-0.25 |
| 3 | Roxithromycin | 0.33 | <0.15 | <0.12 | <0.12 | <0.097 | <0.063 | 0.19 | 0.14 | <0.13 | <0.14 | 0.11 | <0.12 | 0.063-0.2 |
| 3 | Sertraline | 0.17 | 0.28 | <0.12 | <0.084 | <0.11 | <0.07 | <0.14 | <0.16 | <0.14 | <0.088 | <0.093 | <0.12 | 0.07-0.2 |
| 3 | Sotalol | 1.2 | 4 | 0.11 | 6.4 | 0.45 | <0.099 | 0.69 | <0.074 | 0.31 | <0.13 | <0.093 | <0.13 | 0.065-0.14 |
| 3 | Sulfadiazine | <0.19 | <0.17 | 0.35 | <0.3 | <0.2 | <0.15 | <0.14 | <0.16 | <0.16 | <0.25 | <0.21 | <0.3 | 0.13-0.3 |
| 3 | Sulfamerazine | <0.26 | <0.23 | <0.21 | <0.23 | <0.16 | <0.12 | <0.19 | <0.22 | <0.22 | <0.2 | <0.16 | <0.23 | 0.12-0.28 |
| 3 | Sulfamethazine | 0.57 | 2.2 | 1.7 | 0.66 | <0.079 | <0.059 | <0.14 | <0.16 | <0.15 | <0.098 | <0.081 | <0.11 | 0.059-0.2 |
| 3 | Sulfamethizole | 0.46 | 0.94 | <0.2 | <0.23 | <0.16 | <0.12 | <0.19 | <0.21 | <0.21 | <0.2 | <0.16 | <0.23 | 0.12-0.27 |
| 3 | Sulfamethoxazole | 4 | 28 | <0.15 | 12 | <0.27 | <0.19 | 4 | 4.8 | 0.49 | 2.9 | 2.7 | <0.39 | 0.12-0.39 |
| 3 | Sulfapyridine | 2.6 | 13 | 1 | 5.8 | 0.82 | 0.27 | 3.7 | 4.1 | 1.8 | 2.6 | 1.6 | 0.24 | 0.14-0.29 |
| 3 | Tamoxifen | <0.079 | <0.082 | <0.065 | <0.068 | <0.062 | <0.041 | <0.077 | <0.087 | <0.075 | <0.071 | <0.054 | <0.07 | 0.041-0.11 |
| 3 | Telmisartan | 48 | 160 | 5.4 | 200 | 1.5 | <0.26 | 8.6 | 8.8 | 4.1 | 5.3 | 3.6 | <0.49 | 0.16-0.49 |
| 3 | Terbinafine | <0.15 | <0.16 | <0.12 | <0.087 | <0.095 | <0.062 | <0.15 | <0.16 | <0.14 | <0.091 | <0.083 | <0.11 | 0.062-0.21 |
| 3 | Terbutaline | <0.13 | <0.14 | <0.11 | <0.13 | <0.15 | 0.22 | <0.13 | <0.15 | 0.22 | <0.13 | <0.14 | <0.11 | 0.094-0.19 |
| 3 | Tramadol | 13 | 24 | <0.19 | 48 | 0.25 | <0.12 | 4.4 | 5.1 | 0.44 | 3.6 | 2.8 | <0.18 | 0.12-0.32 |
| 3 | Trazodone | <0.17 | <0.18 | <0.14 | <0.12 | <0.1 | <0.087 | <0.17 | <0.19 | <0.16 | <0.11 | <0.082 | <0.12 | 0.082-0.23 |
| 3 | Trimethoprim | 1.3 | 2.8 | <0.16 | 0.85 | <0.13 | <0.099 | 0.83 | 0.59 | <0.16 | 0.42 | 0.23 | <0.19 | 0.099-0.24 |
| 3 | Valsartan | 1.8 | 2.9 | <0.23 | 2 | <0.32 | <0.21 | 4.6 | 5.2 | 0.94 | 0.6 | 0.56 | <0.36 | 0.2-0.39 |
| 3 | Venlafaxine | 3.2 | 2 | <0.16 | 16 | <0.092 | <0.073 | 1.5 | 1.7 | 0.24 | 1.4 | 1.1 | 0.15 | 0.073-0.23 |
| 3 | Verapamil | 0.21 | <0.18 | <0.15 | <0.11 | <0.11 | <0.07 | <0.17 | <0.19 | <0.17 | <0.11 | <0.093 | <0.12 | 0.07-0.25 |
| 3 | Vortioxetine | 0.12 | <0.082 | <0.065 | <0.049 | <0.063 | <0.041 | <0.077 | <0.086 | <0.074 | <0.052 | <0.055 | <0.071 | 0.041-0.11 |
| 3 | Warfarin | <0.32 | 0.75 | <0.36 | 0.95 | <0.19 | <0.15 | <0.29 | <0.28 | <0.28 | <0.42 | <0.2 | <0.25 | 0.15-0.42 |

SM1 Table S5. Target compounds removal efficiency values in percent for two separated treatment sections (Inlet/before GAC and before GAC/Outlets) and for whole DWTPs process (Inlet/Outlet). Heat map visualization highlight the high RE in green colour and low RE in orange and red colour. Negative RE happened when concentration raised during treatment.

|  | **DWTP A spring** | | | **DWTP A autumn** | | | **DWTP B spring** | | | **DWTP B autumn** | | |
| --- | --- | --- | --- | --- | --- | --- | --- | --- | --- | --- | --- | --- |
|  | **I/G** | **G/O** | **I/O** | **I/G** | **G/O** | **I/O** | **I/G** | **G/O** | **I/O** | **I/G** | **G/O** | **I/O** |
| 1H-benzotriazol | -336% | 97% | 85% | 95% | 96% | 100% | -27% | 88% | 85% | 37% | 98% | 98% |
| 1H-benzotriazol_(5/4)-methyl | -383% | 100% | 98% | 100% | 21% | 100% | -8% | 97% | 97% | 59% | 97% | 99% |
| 1H-benzotriazol_1-methyl |  |  |  | -110% | 73% | 44% |  |  |  |  |  |  |
| 2.4-D |  |  |  |  |  |  | -94% | 80% | 61% | 29% | -4% | 26% |
| 4-Isopropylaniline |  |  |  | -192% | 79% | 40% |  |  |  |  |  |  |
| Acetochlor_ESA | -173% | 63% | 0% | 98% | -5% | 98% | -36% | 40% | 18% | 3% | 39% | 41% |
| Acetochlor_OA | -56% | 43% | 10% |  |  |  |  |  |  |  |  |  |
| Alachlor | -31% | 0% | -31% |  |  |  | -33% | 35% | 13% |  |  |  |
| Alachlor_ESA | -206% | 65% | -6% | 89% | 0% | 89% | -48% | 37% | 6% | 57% | 16% | 64% |
| Alachlor_OA | -61% | 53% | 24% |  |  |  | -42% | 31% | 2% |  |  |  |
| Ametryn | 12% | 4% | 15% |  |  |  |  |  |  |  |  |  |
| Anthranilic_acid_isopropylamide | -23% | 32% | 16% | 25% | 17% | 38% |  |  |  |  |  |  |
| Atraton | 14% | 16% | 27% | 19% | 34% | 46% |  |  |  |  |  |  |
| Atrazine | -289% | 92% | 69% | 86% | 83% | 98% | -135% | 90% | 76% | 49% | 56% | 78% |
| Atrazine_2-hydroxy | -173% | 87% | 65% | 74% | 77% | 94% | 0% | 80% | 80% | 18% | 69% | 74% |
| Atrazine_desethyl | -213% | 87% | 58% | 43% | 88% | 93% | -39% | 76% | 67% | 43% | 43% | 68% |
| Atrazine_desisopropyl | -224% | 79% | 32% | 25% | 90% | 92% | -125% | 72% | 38% | -89% | 75% | 54% |
| Azoxystrobin |  |  |  | 47% | -1% | 46% |  |  |  |  |  |  |
| Bentazone | -229% | 95% | 83% | 97% | 0% | 97% | -108% | 60% | 17% | 38% | 51% | 70% |
| Carbendiazim | -4% | 17% | 13% | 53% | 13% | 59% |  |  |  |  |  |  |
| Carbofuran-3-hydroxy | 72% | 38% | 82% | 28% | 62% | 72% | 61% | 0% | 61% | 94% | -35% | 92% |
| Chloridazon_desphenyl | -27% | 42% | 27% | 86% | 15% | 88% | 32% | -12% | 24% | 55% | 6% | 58% |
| Chloridazon_methyl_desphenyl | -136% | 91% | 79% | 89% | 10% | 91% | 9% | 83% | 84% | -7% | 79% | 77% |
| Chlorotoluron | -125% | 97% | 92% | 93% | 27% | 95% | -386% | 97% | 84% | 55% | 81% | 91% |
| Chlorotoluron_desmethyl | 1% | 14% | 15% |  |  |  |  |  |  |  |  |  |
| Clomazone | 9% | 7% | 15% | 71% | 28% | 79% |  |  |  |  |  |  |
| Cyproconazole | -200% | 56% | -31% | 94% | 89% | 99% |  |  |  |  |  |  |
| DEET | -5% | 45% | 43% | 43% | 56% | 75% | -20% | 22% | 7% | 65% | -391% | -73% |
| Diazinon | -528% | 96% | 74% |  |  |  |  |  |  |  |  |  |
| Dimethachlor | -181% | 80% | 44% | 83% | 25% | 87% |  |  |  |  |  |  |
| Dimethachlor_ESA_pos | -93% | 34% | -28% | 93% | 15% | 94% | -43% | 45% | 22% | 83% | -132% | 60% |
| Dimethenamid_ESA |  |  |  |  |  |  | -8% | 45% | 40% | 85% | -43% | 79% |
| Dimethoate | -233% | 78% | 27% | 79% | 29% | 85% | -32% | 49% | 32% |  |  |  |
| Dimethomorph | -16% | 24% | 12% | 72% | 26% | 79% |  |  |  |  |  |  |
| Fenuron |  |  |  | 50% | 13% | 57% |  |  |  |  |  |  |
| Fluazifop-p |  |  |  | 95% | -1% | 95% |  |  |  |  |  |  |
| Hexazinone | -167% | 68% | 13% | 89% | 0% | 89% | -29% | 45% | 29% | 37% | 12% | 44% |
| Imazamox |  |  |  | 54% | 14% | 61% |  |  |  | 72% | 66% | 91% |
| Imidacloprid | -438% | 99% | 96% | 96% | 14% | 97% | 29% | 15% | 39% | 16% | 6% | 21% |
| Isoproturon | -257% | 79% | 25% | 61% | 25% | 71% |  |  |  |  |  |  |
| Isoproturon_monodemethyl |  |  |  | -69% | 64% | 38% |  |  |  |  |  |  |
| Lenacil |  |  |  | -1483% | 68% | -408% |  |  |  |  |  |  |
| MCPA | -18% | 62% | 55% | 62% | 25% | 71% | -13% | 13% | 2% |  |  |  |
| Metalaxyl | -10% | 27% | 20% |  |  |  |  |  |  |  |  |  |
| Metazachlor | -98% | 56% | 14% | 89% | 10% | 90% | -43% | 71% | 58% | 13% | 7% | 19% |
| Metazachlor_OA | 19% | 39% | 51% | 96% | -64% | 93% | -11% | 20% | 11% | 45% | 20% | 56% |
| Methoxyfenozide_pos | -24% | 42% | 29% |  |  |  |  |  |  |  |  |  |
| Metobromuron | 12% | 5% | 16% |  |  |  |  |  |  |  |  |  |
| Metolachlor | -52% | 39% | 7% | 66% | -1% | 66% | -4% | 41% | 39% | 30% | 48% | 64% |
| Metolachlor_ESA | -67% | 56% | 27% | 91% | -24% | 89% | 0% | 49% | 49% | 62% | -61% | 39% |
| Metribuzin_desamino | -14% | 28% | 17% | 61% | 30% | 73% |  |  |  |  |  |  |
| Monolinuron | -18% | 28% | 14% | 27% | 15% | 38% |  |  |  |  |  |  |
| Pirimicarb | 0% | 14% | 14% | 6% | 18% | 22% |  |  |  |  |  |  |
| Propachlor |  |  |  | 16% | 38% | 48% |  |  |  |  |  |  |
| Propazine | -162% | 67% | 14% | 61% | 25% | 71% |  |  |  |  |  |  |
| Propazine_hydroxy | -154% | 81% | 52% | 65% | 73% | 91% | 5% | 75% | 76% | 30% | 37% | 56% |
| Propiconazole | -42% | 65% | 50% | 97% | 88% | 100% | -238% | 77% | 21% | 35% | -27% | 18% |
| Pyrimethanil |  |  |  | 29% | 10% | 36% |  |  |  |  |  |  |
| Sebuthylazine |  |  |  | 0% | -231% | -231% |  |  |  |  |  |  |
| Simazine | -113% | 59% | 13% | 65% | 24% | 73% | -124% | 76% | 45% | 30% | 26% | 48% |
| Simazine_hydroxy | -6% | 21% | 17% | 44% | 16% | 53% |  |  |  |  |  |  |
| Tebuconazole | -72% | 72% | 52% | 90% | 28% | 93% | 42% | 0% | 42% |  |  |  |
| Terbuthylazine | -75% | 32% | -19% | 82% | -59% | 71% | -14% | 47% | 39% | -3% | 57% | 55% |
| Terbuthylazine_desethyl | -83% | -2% | -87% | 16% | 31% | 42% | -32% | 66% | 55% | 35% | 15% | 45% |
| Terbuthylazine_desethyl-2-hydroxy | -48% | 23% | -14% | -127% | 72% | 35% | -44% | 71% | 59% | 7% | 50% | 53% |
| Terbuthylazine_hydroxy | -155% | 86% | 66% | 84% | 68% | 95% | 3% | 79% | 80% | 44% | 72% | 84% |
| Terbutryn | -156% | 73% | 31% | 86% | 25% | 90% | -31% | 18% | -8% | -71% | 41% | 0% |
| Thiamethoxam | -46% | 42% | 15% | 88% | 17% | 90% |  |  |  |  |  |  |
| Triticonazole |  |  |  | 85% | 19% | 88% |  |  |  |  |  |  |
| 10,11-trans-dixydroxy 10,11-dihydroCBZ | -287% | 89% | 57% | 95% | 73% | 99% | -20% | 62% | 55% | -8% | 45% | 41% |
| Alprazolam | -233% | 50% | -67% | 51% | 29% | 65% | -13% | -41% | -60% | -58% | 32% | -8% |
| Atenolol | -144% | 86% | 66% | 85% | 25% | 88% | -43% | 91% | 87% | 11% | 67% | 70% |
| Atorvastatin | -57% | 50% | 21% | 84% | 36% | 90% | -7% | 13% | 7% | -25% | 35% | 19% |
| Bezafibrate |  |  |  |  |  |  | -12% | 0% | -12% |  |  |  |
| Bisoprolol | -333% | 79% | 8% | 80% | 16% | 83% |  |  |  |  |  |  |
| Budenoside | -158% | 66% | 11% |  |  |  |  |  |  |  | 63% |  |
| Caffeine | 18% | 74% | 79% | 91% | -95% | 82% | 17% | 76% | 80% | 21% | 82% | 86% |
| Carbamazepine (CBZ) | -248% | 96% | 85% | 100% | 21% | 100% | -3% | 65% | 63% | 6% | 71% | 73% |
| Carbamazepine 10,11-epoxide | -247% | 90% | 65% | 91% | 73% | 98% | -36% | 34% | 10% | 16% | 29% | 41% |
| Citalopram | -223% | 93% | 77% | 88% | 30% | 92% | -147% | 70% | 26% | -5% | 15% | 11% |
| Clarithromycin | -167% | 99% | 97% | 88% | 15% | 89% | -64% | 66% | 44% | 41% | 44% | 67% |
| Clindamycin | -305% | 96% | 85% | 98% | 15% | 98% | -13% | 73% | 70% | 41% | 67% | 81% |
| Clindamycin_sulfoxide | -253% | 98% | 93% | 99% | 19% | 99% | -33% | 65% | 54% | 1% | 83% | 83% |
| Clonazepam |  |  |  |  |  |  |  |  |  | -7% | 7% | 0% |
| Codeine | -161% | 83% | 55% | 34% | 26% | 51% |  |  |  |  |  |  |
| Diclofenac | -305% | 98% | 93% | 99% | 35% | 99% | -49% | 90% | 85% | -12% | 91% | 90% |
| Erythromycin | -75% | 78% | 62% | 76% | 15% | 79% | 48% | 12% | 55% |  |  |  |
| Fenofibrate | -135% | 65% | 17% |  |  |  | 0% | -100% | -100% |  |  |  |
| Fexofenadine | -346% | 93% | 69% | 98% | 28% | 99% | -12% | 66% | 62% | 36% | 84% | 90% |
| Glimepiride | -71% | 54% | 21% |  |  |  | 32% | 13% | 41% |  |  |  |
| Irbesartan | -355% | 95% | 75% | 96% | 91% | 100% | -17% | 71% | 66% | 12% | 52% | 58% |
| Lamotrigine | -233% | 99% | 98% | 90% | 92% | 99% | -30% | 96% | 95% | -13% | 89% | 88% |
| Memantine | -200% | 73% | 19% | 84% | 28% | 89% |  |  |  |  |  |  |
| Metamphetamine | 24% | -32% | 0% | 72% | 18% | 77% |  |  |  |  |  |  |
| Metoprolol | -381% | 99% | 93% | 99% | 14% | 99% | -739% | 86% | -18% | 43% | 38% | 65% |
| Metoprolol acid | -245% | 94% | 78% | 97% | 56% | 99% | -55% | 89% | 82% | 12% | 87% | 88% |
| Mianserin | -300% | 79% | 17% |  |  |  |  |  |  |  |  |  |
| Mirtazapine | -52% | 81% | 71% | 73% | 27% | 80% |  |  |  |  |  |  |
| N-Desmethylcitalopram |  |  |  | 86% | 31% | 90% |  |  |  |  |  |  |
| O-Desmethylvenlafaxine | 43% | 96% | 98% | 99% | 18% | 99% | -4% | 91% | 91% | -9% | 93% | 92% |
| Oseltamivir carboxylate | 0% | -57% | -57% |  |  |  | -9% | -75% | -91% |  |  |  |
| Oxazepam | -107% | 95% | 91% | 88% | -753% | -4% | 6% | 24% | 28% | -7% | 7% | 0% |
| Oxcarbazepine | 15% | 41% | 50% | 89% | 28% | 92% |  |  |  |  |  |  |
| Propranolol | 44% | 14% | 52% | 100% | 17% | 100% |  |  |  |  |  |  |
| Rosuvastatin | 15% | 62% | 68% | 64% | 32% | 75% | 45% | 12% | 52% | -4% | 0% | -4% |
| Roxithromycin | 55% | 20% | 64% |  |  |  | 26% | 7% | 32% | 21% | -9% | 14% |
| Sertraline | -65% | 57% | 29% |  |  |  |  |  |  |  |  |  |
| Sotalol | -233% | 97% | 91% | 93% | 78% | 98% | 89% | -319% | 55% | 28% | -40% | 0% |
| Sulfadiazine | 11% | -106% | -84% |  |  |  |  |  |  |  |  |  |
| Sulfamethazine | -286% | 23% | -198% | 88% | 25% | 91% |  |  |  |  |  |  |
| Sulfamethizole | -104% | 79% | 57% |  |  |  |  |  |  |  |  |  |
| Sulfamethoxazole | -600% | 99% | 96% | 98% | 30% | 98% | -20% | 90% | 88% | 7% | 86% | 87% |
| Sulfapyridine | -400% | 92% | 62% | 86% | 67% | 95% | -11% | 56% | 51% | 38% | 85% | 91% |
| Telmisartan | -233% | 97% | 89% | 99% | 83% | 100% | -2% | 53% | 52% | 32% | 86% | 91% |
| Terbutaline |  |  |  | -15% | -47% | -69% | -15% | -47% | -69% |  |  |  |
| Tramadol | -85% | 99% | 99% | 99% | 52% | 100% | -16% | 91% | 90% | 22% | 94% | 95% |
| Trimethoprim | -115% | 94% | 88% | 85% | 24% | 88% | 29% | 73% | 81% | 45% | 17% | 55% |
| Valsartan | -61% | 92% | 87% | 84% | 34% | 90% | -13% | 82% | 80% | 7% | 36% | 40% |
| Venlafaxine | 38% | 92% | 95% | 99% | 21% | 100% | -13% | 86% | 84% | 21% | 86% | 89% |
| Verapamil | 14% | 17% | 29% |  |  |  |  |  |  |  |  |  |
| Vortioxetine | 32% | 21% | 46% |  |  |  |  |  |  |  |  |  |
| Warfarin | -134% | 52% | -13% | 80% | 21% | 84% |  |  |  |  |  |  |

| \| SM1 Table S6. Detected compound numbers and summed peak areas \| \| \| \| \| \| \| \| --- \| --- \| --- \| --- \| --- \| --- \| --- \| \|  \|  \|  \|  \| \|  \| \|  \|  \| **Sampling sites** \| **Number of compounds** \| \| **Sum of Areas** \| \| DWTP A \| spring \| Inlet \| 1 003 \| \| 1.6.10^10^ \| \|  \|  \| before GAC \| \| 977 \| 1.6.10^10^ \| \|  \|  \| Outlet \| 935 \| \| 1.5.10^10^ \| \|  \| autumn \| Inlet \| 1 275 \| \| 2.5.10^10^ \| \|  \|  \| before GAC \| 928 \| \| 2.1.10^10^ \| \|  \|  \| Outlet \| 1 184 \| \| 3.2.10^10^ \| \| DWTP B \| spring \| Inlet \| 719 \| \| 1.4.10^10^ \| \|  \|  \| before GAC \| 849 \| \| 1.5.10^10^ \| \|  \|  \| Outlet \| 782 \| \| 1.3.10^10^ \| \|  \| autumn \| Inlet \| 722 \| \| 1.4.10^10^ \| \|  \|  \| before GAC \| 693 \| \| 1.4.10^10^ \| \|  \|  \| Outlet \| 630 \| \| 1.4.10^10^ \| |
| --- | --- | --- | --- | --- | --- | --- | --- | --- | --- | --- | --- | --- | --- | --- | --- | --- | --- | --- | --- | --- | --- | --- | --- | --- | --- | --- | --- | --- | --- | --- | --- | --- | --- | --- | --- | --- | --- | --- | --- | --- | --- | --- | --- | --- | --- | --- | --- | --- | --- | --- | --- | --- | --- | --- | --- | --- | --- | --- | --- | --- | --- | --- | --- | --- | --- | --- | --- | --- | --- | --- | --- | --- | --- | --- | --- | --- | --- | --- | --- | --- | --- | --- | --- | --- | --- | --- | --- | --- | --- | --- | --- |

SM1 Table S7. Compound´s numbers and summed areas for each log2FC level in treatment sections.

I = Inlet, b GAC = before GAC, O = Outlet. Log2FC levels correspond with compounds changes in treatment

in the following order: no change, downregulated, eliminated, upregulated, formed.

|  |  | Change | no change | Decreased | Decreased | Eliminated | Eliminated | Increased | Increased | Formed | Formed |
| --- | --- | --- | --- | --- | --- | --- | --- | --- | --- | --- | --- |
|  |  | log2FC  level | between  1 and -1 | >1 | >1 | Infinity | Infinity | < -1 | < -1 | -Infinity | -Infinity |
|  |  | Value | Numbers | Numbers | Areas | Numbers | Areas | Numbers | Areas | Numbers | Areas |
| Season | Location | Treatment  section |  |  |  |  |  |  |  |  |  |
| Spring | DWTP A | I / O | 389 | 34 | 5.02E+08 | 536 | 3.77E+09 | 44 | 8.31E+08 | 468 | 3.66E+09 |
|  |  | I / b GAC | 368 | 25 | 4.04E+08 | 540 | 4.67E+09 | 70 | 1.68E+09 | 514 | 3.56E+09 |
|  |  | b GAC / O | 308 | 53 | 1.62E+09 | 572 | 4.14E+09 | 44 | 9.53E+08 | 530 | 4.59E+09 |
|  |  |  |  |  |  |  |  |  |  |  |  |
| Autumn | DWTP A | I / O | 233 | 50 | 1.94E+09 | 925 | 9.92E+09 | 67 | 9.56E+09 | 834 | 9.70E+09 |
|  |  | I / b GAC | 281 | 45 | 1.62E+09 | 914 | 1.11E+10 | 35 | 2.00E+09 | 567 | 4.92E+09 |
|  |  | b GAC / O | 375 | 34 | 3.32E+08 | 414 | 7.58E+09 | 105 | 7.44E+09 | 670 | 1.11E+10 |
|  |  |  |  |  |  |  |  |  |  |  |  |
| Spring | DWTP B | I / O | 233 | 50 | 1.62E+09 | 404 | 3.24E+09 | 32 | 5.94E+08 | 467 | 3.31E+09 |
|  |  | I / b GAC | 313 | 16 | 8.87E+08 | 361 | 3.74E+09 | 29 | 5.11E+08 | 491 | 5.80E+09 |
|  |  | b GAC / O | 270 | 58 | 1.84E+09 | 488 | 4.21E+09 | 33 | 6.44E+08 | 421 | 3.25E+09 |
|  |  |  |  |  |  |  |  |  |  |  |  |
| Autumn | DWTP B | I / O | 245 | 33 | 3.00E+08 | 437 | 4.42E+09 | 7 | 9.34E+07 | 345 | 3.63E+09 |
|  |  | I / b GAC | 362 | 17 | 5.08E+08 | 395 | 3.27E+09 | 8 | 5.38E+07 | 306 | 3.25E+09 |
|  |  | b GAC / O | 251 | 36 | 8.86E+08 | 389 | 3.98E+09 | 17 | 6.79E+08 | 326 | 3.39E+09 |

SM1 Table S8. Compound´s with Chlorine and Bromine isotopic pattern numbers and summed areas for each log2FC level

in treatment sections.

|  |  | Change | no change | Decreased | Decreased | Eliminated | Eliminated | Increased | Increased | Formed | Formed |
| --- | --- | --- | --- | --- | --- | --- | --- | --- | --- | --- | --- |
|  |  | log2FC  level | between  1 and -1 | >1 | >1 | Infinity | Infinity | < -1 | < -1 | -Infinity | -Infinity |
|  |  | Value | Numbers | Numbers | Areas | Numbers | Areas | Numbers | Areas | Numbers | Areas |
| Season | Location | Treatment  section |  |  |  |  |  |  |  |  |  |
| Spring | DWTP A | I / O | 72 | 7 | 6.22E+07 | 53 | 1.30E+08 | 6 | 2.45E+07 | 109 | 1.76E+08 |
|  |  | I / b GAC | 54 | 5 | 8.10E+07 | 64 | 1.38E+08 | 15 | 1.44E+08 | 54 | 1.06E+08 |
|  |  | b GAC / O | 50 | 9 | 5.38E+07 | 61 | 2.80E+08 | 8 | 5.15E+07 | 127 | 1.99E+08 |
|  |  |  |  |  |  |  |  |  |  |  |  |
| Autumn | DWTP A | I / O | 40 | 10 | 5.05E+07 | 125 | 4.68E+08 | 7 | 1.72E+08 | 116 | 1.72E+08 |
|  |  | I / b GAC | 45 | 13 | 3.22E+08 | 121 | 1.95E+08 | 3 | 7.58E+06 | 43 | 7.34E+07 |
|  |  | b GAC / O | 47 | 11 | 2.99E+07 | 37 | 6.40E+07 | 9 | 2.17E+08 | 106 | 1.53E+08 |
|  |  |  |  |  |  |  |  |  |  |  |  |
| Spring | DWTP B | I / O | 30 | 10 | 7.82E+07 | 54 | 5.51E+07 | 1 | 0.00E+00 | 53 | 3.62E+08 |
|  |  | I / b GAC | 56 | 2 | 3.28E+07 | 36 | 3.21E+07 | 0 | 0.00E+00 | 95 | 4.34E+08 |
|  |  | b GAC / O | 41 | 8 | 1.42E+08 | 102 | 2.05E+08 | 2 | 1.27E+07 | 41 | 5.95E+07 |
|  |  |  |  |  |  |  |  |  |  |  |  |
| Autumn | DWTP B | I / O | 26 | 3 | 1.42E+06 | 54 | 7.24E+07 | 0 | 4.61E+05 | 31 | 2.19E+07 |
|  |  | I / b GAC | 58 | 1 | 5.32E+06 | 25 | 3.52E+07 | 0 | 0.00E+00 | 18 | 1.24E+07 |
|  |  | b GAC / O | 25 | 7 | 6.69E+07 | 44 | 4.63E+07 | 2 | 1.89E+06 | 28 | 1.96E+07 |

I = Inlet, b GAC = before GAC, O = Outlet. Log2FC levels correspond with compounds changes in treatment in the following order: no change, downregulated, eliminated, upregulated, formed.
