# Supplementary material for: Novel nontarget LC-HRMS-based approaches for evaluation of drinking water treatment
Source: Environ Monit Assess. 2023 May 26;195(6):739. doi: 10.1007/s10661-023-11348-w (PMC10219882; doi:10.1007/s10661-023-11348-w)
Supplement: Supplementary file 2 — Supplementary file2 (DOCX 76 KB) [file 10661_2023_11348_MOESM2_ESM.docx]

**Novel nontarget LC-HRMS-based approaches for evaluation of drinking water treatment**

**Supplementary materials 2 – description of analytical methods**

Petra Nováková^a^*, Helena Švecová^a^, Adam Bořík^a^, Roman Grabic^a^

*^a^University of South Bohemia in České Budějovice, Faculty of Fisheries and Protection of Waters, South Bohemian Research Centre of Aquaculture and Biodiversity of Hydrocenoses, Zátiší 728/II, CZ-389 25, Vodňany, Czech Republic*

* Corresponding author. Tel.: +420 38777 4752; E-mail address: novakovapetra@frov.jcu.cz; Faculty of Fisheries and Protection of Waters, Zátiší 728/II, 389 25 Vodňany, Czech Republic

SM2 Table S1. Reference standards information ^1^

| **Target analyte** | **Standard producer** | **Polarity** | **Precursor (*m/z*)** | **Quan (*m/z*)** | **Collision En. (V)** | **Qual (m/z)** | **Collision En. (V)** | **RF Lens (V)** | **Retention Time (min)** | **IS_used** |
| --- | --- | --- | --- | --- | --- | --- | --- | --- | --- | --- |
| 1-(3,4-Dichlorophenyl)_urea | Dr.Ehrenstorfer | - | 202.97 | 159.946 | 14.045 | 124.014 | 20.921 | 52.247 | 6.6 | 2,4-D_IS |
| Dihydro CBZ | Fluka | + | 239.07 | 194.071 | 23.904 | 180.071 | 39.831 | 65 | 7.6 | Carbamazepine_IS |
| trans-dihydro-dixydroxy CBZ | TRC | + | 271.087 | 236 | 12.174 | 180.071 | 29.517 | 35 | 5.9 | Carbamazepine_IS |
| 1H-benzotriazol | Sigma-Aldrich | + | 120.017 | 65.111 | 22.59 | 92.093 | 17.837 | 58 | 3.9 | Carbendazim_IS |
| 1H-benzotriazol_(5-4)-methyl | Sigma-Aldrich | + | 134.048 | 79.183 | 20 | 106.111 | 17 | 58 | 5 | Trimethoprim_IS |
| 1H-benzotriazol_1-methyl | Sigma-Aldrich | + | 133.957 | 77.111 | 23.904 | 79.111 | 19.354 | 57 | 4.8 | Trimethoprim_IS |
| 2,4,5-trichlorophenoxyacetic_acid | Sigma-Aldrich | - | 252.961 | 194.889 | 13.034 | 158.972 | 28.506 | 30 | 7.7 | MCPP_IS |
| 2,4-D | Sigma-Aldrich | - | 219.052 | 160.946 | 13.539 | 125 | 28.404 | 35.685 | 7.2 | 2,4-D_IS |
| 2,4-D_IS (13C6) | CIL | - | 225.052 | 166.942 | 10.253 | 131.026 | 28.253 | 34.697 | 7.17 | - |
| 2,4-Dichlorphenoxypropionic_acid | Chromservice | - | 233 | 160.929 | 11.517 | 124.986 | 28.961 | 32.225 | 7.7 | MCPP_IS |
| 3-chloro-4-methylaniline | TRC | + | 142.293 | 107.111 | 18.242 | 106.111 | 27.747 | 52.247 | 4.2 | Trimethoprim_IS |
| 4-Isopropylaniline | Sigma-Aldrich | + | 177.222 | 136.183 | 10.253 | 94.04 | 24.916 | 48.292 | 4.8 | Trimethoprim_IS |
| Acetochlor | Chromservice | + | 224.05 | 148.111 | 16.624 | 133.111 | 28.051 | 49.528 | 8.6 | Alachlor_IS |
| Acetochlor_ESA | Sigma-Aldrich | - | 314.13 | 162.111 | 24.36 | 121 | 23.298 | 90.809 | 5.4 | 2,4-D_IS |
| Acetochlor_OA | HPC | - | 264.161 | 146.111 | 10.253 | 144.097 | 28.202 | 30 | 5.9 | 2,4-D_IS |
| Alachlor | Chromservice | + | 238.1 | 162.111 | 17.989 | 132.111 | 37.758 | 58.674 | 8.6 | Alachlor_IS |
| Alachlor_ESA | HPC | - | 314.161 | 149.111 | 26.382 | 160.125 | 24.056 | 88.584 | 5.4 | Metazachlor_ESA_IS |
| Alachlor_IS | Dr. Ehrenstorfer | + | 283.243 | 175.222 | 20.669 | 251.111 | 12.225 | 57 | 8.6 | - |
| Alachlor_OA | Sigma-Aldrich | - | 264.161 | 160.111 | 11.264 | 158.111 | 26.837 | 30.494 | 6.1 | 2,4-D_IS |
| Alfuzosin | AK Scientific | + | 390.23 | 156.111 | 26.989 | 235.04 | 27.242 | 93 | 6.5 | Tramadol_IS |
| Alprazolam | Chiron | + | 309.109 | 281 | 25.927 | 205 | 41.904 | 89 | 8.3 | Carbamazepine_IS |
| Ametryn | Chromservice | + | 228.13 | 186.111 | 19.101 | 96.111 | 26.888 | 63.124 | 6.2 | Simazine_IS |
| Amitryptyline | Sigma-Aldrich | + | 278.178 | 233.04 | 17.483 | 191.071 | 25.674 | 63 | 9.4 | Amitryptyline_IS |
| Amitryptyline_IS (D6) | CDN Isotopes | + | 284.23 | 233.04 | 17.635 | - | - | 64 | 9.4 | - |
| Anthranilic_acid_isopropylamide | Sigma-Aldrich | + | 179.13 | 120.058 | 14.854 | 92.111 | 29.163 | 30 | 3.8 | Carbendazim_IS |
| Atenolol | Sigma-Aldrich | + | 267.15 | 145.04 | 26.584 | 190.071 | 18.393 | 63 | 4.5 | Atenolol_IS |
| Atenolol_IS (D6) | Alsa Chim | + | 273.283 | 145.054 | 26.584 | - | - | 63 | 4.5 | - |
| Atorvastatin | Chemos GmbH | + | 559.261 | 440.222 | 20.264 | 466.236 | 14.197 | 86 | 9.5 | Carbamazepine_IS |
| Atraton | Sigma-Aldrich | + | 212.222 | 170.111 | 19.404 | 100.04 | 29.264 | 64.112 | 5.2 | Trimethoprim_IS |
| Atrazine | Chem Servise | + | 216.172 | 174.058 | 18.747 | 104 | 30.073 | 58.427 | 6.8 | Atrazine_IS |
| Atrazine_2-hydroxy | Neochema | + | 198.404 | 156.111 | 17.837 | 86.04 | 24.208 | 60.404 | 4.2 | Carbendazim_IS |
| Atrazine_desethyl | Neochema | + | 188.04 | 146.071 | 18.14 | 103.986 | 27.292 | 60.899 | 4.7 | Trimethoprim_IS |
| Atrazine_desethyl-2-hydroxy | Dr. Ehrenstorfer | + | 170.202 | 128.04 | 17.079 | 86.058 | 24.663 | 52.494 | 1.5 | Carbendazim_IS |
| Atrazine_desethyl-desisopropyl | Neochema | + | 145.928 | 104 | 20.315 | 79.071 | 20.011 | 43.843 | 1.3 | Carbendazim_IS |
| Atrazine_desisopropyl | Neochema | + | 174.314 | 104 | 24.461 | 132 | 17.989 | 59.169 | 3.9 | Carbendazim_IS |
| Atrazine_IS (D5) | Chiron | + | 221.152 | 179.04 | 18.949 | 137.04 | 24.36 | 66 | 6.7 | - |
| Azithromycin | AK Scientific | + | 749.53 | 591.458 | 27.747 | 573.387 | 32.702 | 104 | 7.5 | Clarithromycin_IS |
| Azoxystrobin | HPC | + | 404.13 | 372.04 | 14.146 | 329.071 | 29.921 | 58 | 8.2 | Trimethoprim_IS |
| Bensulfuron_methyl | Sigma-Aldrich | + | 411.08 | 149.04 | 20.82 | 182.054 | 20.011 | 66.337 | 7.6 | Terbuthylazine_IS |
| Bentazone | Sigma-Aldrich | - | 239.08 | 132.071 | 28.202 | 197 | 22.135 | 75.978 | 6.8 | 2,4-D_IS |
| Bezafibrate | Sigma-Aldrich | + | 362.13 | 139 | 25.624 | 121.04 | 28.708 | 59 | 8.8 | Carbamazepine_IS |
| Biperiden | TRC | + | 312.291 | 98.111 | 22.944 | 294.111 | 15.916 | 60 | 8.9 | Carbamazepine_IS |
| Bisoprolol | AK Scientific | + | 326.23 | 116.111 | 17.281 | 74.111 | 26.079 | 72 | 7.1 | Metoprolol_IS |
| Budenoside | Sigma-Aldrich | + | 431.2 | 413.4 |  |  | 7 | 116 | 9.6 |  |
| Caffeine | HPC | + | 195.152 | 138.04 | 19.758 | 110.111 | 23.551 | 62 | 4.9 | Caffeine_IS |
| Caffeine_IS (13C6) | TRC | + | 198.1 | 140.111 | 19.86 | - | - | 62 | 4.9 | - |
| Carbamazepine (CBZ) | Sigma-Aldrich | + | 237.07 | 194.054 | 20.213 | 192.04 | 24.562 | 61 | 7.5 | Carbamazepine_IS |
| Carbamazepine_IS (D10) | Chiron | + | 247.222 | 204.111 | 21.579 | - | - | 64 | 7.5 | - |
| Epoxy CBZ | TRC | + | 253.009 | 180.071 | 28.404 | 182.058 | 24.258 | 46 | 6.6 | Carbamazepine_IS |
| Carbendazim | Sigma-Aldrich | + | 192.111 | 160.071 | 18.798 | 132.071 | 31.792 | 52.989 | 3.3 | Carbendazim_IS |
| Carbendazim_IS (D3) | Chiron | + | 195.152 | 160.058 | 19.506 | 132.04 | 31.084 | 57 | 3.2 | - |
| Carbofuran-3-hydroxy | Sigma-Aldrich | + | 163.324 | 107.058 | 21.478 | 135.111 | 14.298 | 56.449 | 4.7 | Trimethoprim_IS |
| Cetirizine | Sigma-Aldrich | + | 389.17 | 201 | 18.292 | 165.054 | 55 | 57 | 9.1 | Carbamazepine_IS |
| Chlorantraniliprole | Sigma-Aldrich | + | 483.991 | 452.889 | 18.646 | 285.889 | 13.742 | 62.876 | 7.6 | Simazine_IS |
| Chloridazon | Sigma-Aldrich | + | 222.05 | 104.111 | 23.197 | 146.054 | 27.494 | 69 | 4.8 | Simazine_IS |
| Chloridazon_desphenyl | Dr. Ehrenstorfer | + | 145.98 | 117 | 23.601 | 66.111 | 38.466 | 58.921 | 1.3 | Carbendazim_IS |
| Chloridazon_methyl_desphenyl | HPC | + | 160.009 | 88.054 | 32.045 | 117 | 24.258 | 64 | 3.3 | Carbendazim_IS |
| Chlorotoluron | HPC | + | 213.1 | 72.111 | 19.91 | 46.222 | 17.938 | 54.472 | 6.9 | Simazine_IS |
| Chlorotoluron_desmethyl | HPC | + | 198.989 | 107.111 | 24.511 | 142.054 | 15.36 | 48 | 6.7 | Simazine_IS |
| Chlorpyrifos | Sigma-Aldrich | + | 351.939 | 199.889 | 21.174 | 323.889 | 10.253 | 57.933 | 10.1 | Alachlor_IS |
| Cilazapril | EDQM | + | 418.261 | 211.058 | 18.393 | 70.111 | 41.197 | 59 | 8.7 | Carbamazepine_IS |
| Citalopram | AK Scientific | + | 325.2 | 109.058 | 27.09 | 262.071 | 19.303 | 72 | 8.5 | Citalopram_IS |
| Citalopram_IS (D6) | TRC | + | 331.23 | 262.04 | 20 | - | - | 78 | 8.5 | - |
| Clarithromycin | Sigma-Aldrich | + | 748.461 | 590.333 | 17.23 | 558.405 | 20.618 | 83 | 9.1 | Clarithromycin_IS |
| Clarithromycin_IS (D3) | TRC | + | 751.5 | 593.405 | 17.028 | - | - | 84 | 9 | - |
| Clemastine | AK Scientific | + | 344.252 | 215 | 16.826 | 130.183 | 10.253 | 45 | 10.2 | Carbamazepine_IS |
| Clindamycin | TRC | + | 425.23 | 126.111 | 27.798 | 377.111 | 17.938 | 75 | 7 | Clindamycin_IS |
| Clindamycin_IS (D3) | TRC | + | 428.2 | 129.183 | 28.404 | - | - | 76 | 7 | - |
| Clindamycin_sulfoxide | Sigma-Aldrich | + | 441.139 | 126.169 | 30.073 | 377.111 | 15.815 | 61 | 6 | Clindamycin_IS |
| Clomazone | Sigma-Aldrich | + | 240.05 | 125 | 22.994 | 89.04 | 49.59 | 50.764 | 7.5 | Simazine_IS |
| Clomipramine | Sigma-Aldrich | + | 315.211 | 86.111 | 17.787 | 227 | 42 | 58 | 8.41 | Carbamazepine_IS |
| Clonazepam | Chiron | + | 316.061 | 214 | 38.062 | 270 | 38.062 | 83 | 8.1 | Oxazepam_IS |
| Codeine | Chiron | + | 300.191 | 165.058 | 41.5 | 215.04 | 25.118 | 75 | 5 | Atenolol_IS |
| Cyproconazole | Sigma-Aldrich | + | 292.161 | 70.111 | 21.528 | 125 | 30.983 | 57.191 | 7.9 | Simazine_IS |
| DEET | Fluka | + | 192.13 | 119.04 | 18.242 | 91.111 | 31.034 | 56.449 | 7 | Simazine_IS |
| Desmetryn | Sigma-Aldrich | + | 214.1 | 172.054 | 18.798 | 82.111 | 32.551 | 57.685 | 5.45 | Simazine_IS |
| Diazinon | Absolute Standard | + | 305.111 | 169.111 | 21.68 | 153.111 | 21.68 | 67.573 | 9.1 | Alachlor_IS |
| Diclofenac | Sigma-Aldrich | + | 296.01 | 214 | 34.725 | 215 | 19.2 | 48 | 9.5 | Diclofenac_IS |
| Diclofenac_IS (13C6) | Chiron | + | 302.07 | 220.058 | 32.753 | - | - | 50 | 9.5 | - |
| Dicycloverine | EDQM | + | 310.291 | 165.111 | 19.253 | 237.111 | 19.556 | 61 | 10.5 | Carbamazepine_IS |
| Diltiazem | Sigma-Aldrich | + | 415.18 | 178 | 24.056 | 150.071 | 42.056 | 74 | 8.5 | Carbamazepine_IS |
| Dimethachlor | HPC | + | 256.13 | 224 | 16.169 | 148.111 | 26.382 | 43.596 | 7.4 | Simazine_IS |
| Dimethachlor_ESA | HPC | + | 302.13 | 270.04 | 13.994 | 132.04 | 34.624 | 50 | 4.3 | Simazine_IS |
| Dimethachlor_OA | HPC | + | 252.161 | 220.111 | 12.528 | 132.111 | 24.056 | 39 | 4.9 | Simazine_IS |
| Dimethenamid_ESA | Chem Servise | + | 322.08 | 290 | 13.539 | 210.04 | 21.124 | 49 | 4.8 | Simazine_IS |
| Dimethenamid_OA | Chem Servise | + | 272.1 | 239.982 | 12.376 | 126.071 | 30.225 | 38 | 5.5 | Simazine_IS |
| Dimethoate | Sigma-Aldrich | + | 199.052 | 125 | 17.837 | 170.946 | 10.253 | 70.787 | 4.7 | Simazine_IS |
| Dimethomorph | Chromservice | + | 388.161 | 301 | 19.708 | 165.125 | 30.579 | 79 | 7.7 | Terbuthylazine_IS |
| Diphenhydramine | Sigma-Aldrich | + | 256.15 | 167.04 | 12.68 | 165.071 | 40.792 | 35 | 8.3 | Carbamazepine_IS |
| Disopyramide | Sigma-Aldrich | + | 340.23 | 239.111 | 17.079 | 195.071 | 29.365 | 58 | 6.7 | Tramadol_IS |
| Diuron | Chromservice | + | 233.02 | 72.111 | 20.77 | 46.222 | 18.444 | 53.236 | 7.2 | Simazine_IS |
| Diuron_desmethyl | Chromservice | + | 219 | 127.071 | 27.393 | 162 | 14.854 | 58 | 6.9 | Simazine_IS |
| Donepezil | Sigma-Aldrich | + | 380.291 | 91.04 | 35.787 | 243.111 | 26.079 | 83 | 7.9 | Tramadol_IS |
| Epoxiconazole | Sigma-Aldrich | + | 330.1 | 121.04 | 23.803 | 123.071 | 20.264 | 63.371 | 8.3 | Terbuthylazine_IS |
| Erythromycin | Sigma-Aldrich | + | 734.491 | 576.405 | 17.433 | 558.405 | 16.725 | 80 | 8 | Clindamycin_IS |
| Fenofibrate | Sigma-Aldrich | + | 361.161 | 232.929 | 16.017 | 139 | 28.253 | 59 | 10.8 | Carbamazepine_IS |
| Fenuron | Sigma-Aldrich | + | 165.039 | 72.111 | 18.848 | 46.222 | 15.663 | 56.202 | 4.2 | Carbendazim_IS |
| Fexofenadine | TRC | + | 502.27 | 466.222 | 26.483 | 484.276 | 22.084 | 89 | 9.3 | Carbamazepine_IS |
| Florasulam | Sigma-Aldrich | + | 360.05 | 129.058 | 25.522 | 192.014 | 16.573 | 69.798 | 6.7 | Simazine_IS |
| Fluazifop-p | Sigma-Aldrich | + | 328.09 | 282.071 | 18.393 | 254 | 26.635 | 73 | 8 | Terbuthylazine_IS |
| Flusilazole | Sigma-Aldrich | + | 316.05 | 247 | 18.545 | 165.04 | 29.871 | 69.056 | 8.6 | Alachlor_IS |
| Foramsulfuron | Sigma-Aldrich | + | 453.1 | 182.058 | 21.427 | 272 | 12.831 | 63.371 | 6.4 | Simazine_IS |
| Gabapentin | Sigma-Aldrich | + | 172.15 | 137.11 | 17 | 154.11 | 14 | 41 | 4.8 | Trimethoprim_IS |
| Glibenclamide | Sigma-Aldrich | + | 494.18 | 369 | 13.489 | 169 | 33.663 | 57 | 9.5 | Carbamazepine_IS |
| Glimepiride | TRC | + | 491.261 | 352.04 | 12.022 | 126.111 | 24.966 | 57 | 9.6 | Carbamazepine_IS |
| Haloperidol | Sigma-Aldrich | + | 376.13 | 165.054 | 23.045 | 123.071 | 38.011 | 76 | 8.8 | Carbamazepine_IS |
| Hexazinone | AccuStandard | + | 253.15 | 171.111 | 16.674 | 71.097 | 33.612 | 47.303 | 5.9 | Simazine_IS |
| Imazamethabenz_methyl | Sigma-Aldrich | + | 289.161 | 144.058 | 38.82 | 229.111 | 20.466 | 64.112 | 5.7 | Simazine_IS |
| Imazamox | Sigma-Aldrich | + | 306.161 | 261.111 | 21.174 | 193.054 | 27.343 | 67.326 | 4.9 | Carbendazim_IS |
| Imidacloprid | Sigma-Aldrich | + | 256.081 | 175.111 | 18.899 | 209 | 17.382 | 45.573 | 4.9 | Carbendazim_IS |
| Ioxynil | Chromservice | - | 369.83 | 126.875 | 40.994 | 214.889 | 35.331 | 81.663 | 7.7 | 2,4-D_IS |
| Irbesartan | TRC | + | 429.261 | 207.04 | 23.197 | 195.111 | 21.528 | 73 | 8.8 | Carbamazepine_IS |
| Isoproturon | Chromservice | + | 207.11 | 72.111 | 20.315 | 46.151 | 19.404 | 53.236 | 7.1 | Atrazine_IS |
| Isoproturon_didemethyl | Chromservice | + | 179.13 | 137.111 | 12.983 | 94.111 | 21.68 | 41 | 6.6 | Simazine_IS |
| Isoproturon_monodemethyl | Chromservice | + | 193.171 | 151.111 | 12.831 | 94.111 | 20.921 | 48 | 6.8 | Simazine_IS |
| Lamotrigine | Sigma-Aldrich | + | 256.03 | 159 | 29 | 210.929 | 27 | 85 | 6.4 | Carbamazepine_IS |
| Lenacil | Chromservice | + | 235.13 | 153.04 | 17.028 | 136.04 | 33.713 | 33.213 | 6.4 | Simazine_IS |
| Linuron | Chromservice | + | 249.02 | 160.014 | 19.506 | 182 | 17.331 | 54.719 | 7.9 | Metolachlor_IS |
| Loperamide | Sigma-Aldrich | + | 477.261 | 266.111 | 24.056 | 210.111 | 47.82 | 78 | 10.4 | Carbamazepine_IS |
| Malathion | Sigma-Aldrich | + | 331.061 | 99 | 22.742 | 127.071 | 12.68 | 38.899 | 8.6 | Metolachlor_IS |
| Maprotiline | Sigma-Aldrich | + | 278.23 | 250.111 | 18.393 | 191.071 | 35.888 | 61 | 9.3 | Amitryptyline_IS |
| MCPA | Lipomed | - | 199.02 | 141 | 15.208 | 155.014 | 10.253 | 39.888 | 7.27 | MCPP_IS |
| MCPP | Chromservice | - | 213.03 | 141.071 | 14.702 | 71.083 | 10.253 | 30.989 | 7.7 | MCPP_IS |
| MCPP_IS (D3) | Chiron | - | 216.243 | 144.111 | 11.77 | 172.125 | 11.213 | 49 | 7.7 | - |
| Meclozine | EDQM | + | 391.252 | 201 | 16.876 | 166.071 | 37.961 | 55 | 10.1 | Carbamazepine_IS |
| Memantine | AK Scientific | + | 180.099 | 163.111 | 16.32 | 107.111 | 26.433 | 30 | 7.3 | Tramadol_IS |
| Metalaxyl | Absolute Standard | + | 280.13 | 220.111 | 13.742 | 160.111 | 24.966 | 47.798 | 7.1 | Atrazine_IS |
| Metamphetamine | Sigma-Aldrich | + | 150.11 | 91.111 | 20.567 | 119.111 | 10.253 | 39.888 | 5.3 | Metamphetamine_IS |
| Metamphetamine_IS (D5) | Chiron | + | 155.222 | 92.111 | 20.466 |  |  | 38.899 | 5.3 | - |
| Metazachlor | Absolute Standard | + | 278.131 | 134.111 | 23.601 | 210.071 | 10.253 | 37.169 | 7.3 | Simazine_IS |
| Metazachlor_ESA | HPC | - | 322.1 | 121.143 | 22.135 | 148.183 | 23.955 | 65 | 4.5 | Metazachlor_ESA_IS |
| Metazachlor_ESA_IS (D5) | HPC | - | 328.13 | 121 | 22.539 | 154.111 | 25.371 | 88 | 4.9 | - |
| Metazachlor_OA | Chiron | + | 274.13 | 134.111 | 19.455 | 162.111 | 10.253 | 33 | 4.5 | Metolachlor_IS |
| Metconazole | Sigma-Aldrich | + | 320.161 | 70.111 | 26.23 | 125 | 37.809 | 59.663 | 8.6 | Metolachlor_IS |
| Methabenzthiazuron | Chromservice | + | 222.009 | 165.071 | 18.444 | 150 | 36.14 | 45.079 | 6.8 | Simazine_IS |
| Methoxyfenozide | Chem Servise | + | 369.23 | 149.04 | 17.129 | 313.04 | 10.253 | 35 | 8.5 | Metolachlor_IS |
| Metobromuron | Chromservice | + | 258.98 | 169.942 | 21.073 | 148.111 | 16.169 | 52.247 | 7.3 | Simazine_IS |
| Metolachlor | Chromservice | + | 284.161 | 252.058 | 15.663 | 176.111 | 27.242 | 47.056 | 8.6 | Metolachlor_IS |
| Metolachlor_ESA | Sigma-Aldrich | + | 330.13 | 298.04 | 15.107 | 202.111 | 30.124 | 56.944 | 5.4 | Carbendazim_IS |
| Metolachlor_IS (13C6) | CIL | + | 290.13 | 258.04 | 15.815 | 182.111 | 27.292 | 48.539 | 8.5 | - |
| Metolachlor_OA | Chem Servise | + | 280.191 | 248.04 | 13.337 | 131.04 | 32.197 | 42 | 7 | Simazine_IS |
| Metoprolol | Sigma-Aldrich | + | 268.17 | 116.111 | 18.494 | 191.111 | 17.787 | 63 | 6.3 | Metoprolol_IS |
| Metoprolol acid | TRC | + | 268.435 | 145.129 | 24.157 | 190.911 | 15.713 | 64 | 5.2 | Metoprolol_IS |
| Metoprolol_IS (D7) | Alsa Chim | + | 275.23 | 123.169 | 19.556 | - | - | 64 | 6.3 | - |
| Metoxuron | Chromservice | + | 229.08 | 72.04 | 20.719 | 46.165 | 18.798 | 51.753 | 5.8 | Simazine_IS |
| Metribuzin | Chem Servise | + | 215.039 | 187.111 | 18.646 | 131.071 | 22.742 | 59.416 | 5.9 | Simazine_IS |
| Metribuzin_desamino | Dr. Ehrenstorfer | + | 200.161 | 172.111 | 18.09 | 116.071 | 22.843 | 53 | 5.7 | Simazine_IS |
| Metsulfuron-methyl | Sigma-Aldrich | + | 382.08 | 167.04 | 16.472 | 141.111 | 16.371 | 58.674 | 6.7 | Simazine_IS |
| Mianserin | AK Scientific | + | 265.18 | 208.04 | 20.669 | 263.111 | 19.607 | 64 | 8.3 | Carbamazepine_IS |
| Miconazole | Sigma-Aldrich | + | 417.052 | 159 | 29.77 | 161 | 30.629 | 91 | 10.2 | Carbamazepine_IS |
| Mirtazapine | AK Scientific | + | 266.211 | 195.054 | 26.129 | 209.111 | 20.77 | 61 | 6 | Tramadol_IS |
| Monolinuron | Chromservice | + | 215.324 | 126 | 17.888 | 148.058 | 14.652 | 46.315 | 7.1 | Simazine_IS |
| N1_AcetylSMX | Sigma-Aldrich | + | 296.03 | 254 | 10.253 | 123.071 | 10.253 | 34 | 7.4 | Sulfamethoxazole_IS |
| N4_AcetylSMX | Sigma-Aldrich | + | 296.061 | 198 | 16.775 | 134.054 | 22.944 | 64 | 6.5 | Sulfamethoxazole_IS |
| N-chloroacetyl-2,6-diethylaniline | Dr. Ehrenstorfer | + | 226.08 | 170 | 22.388 | 198.054 | 17.23 | 57.438 | 6 | Simazine_IS |
| N-Desmethylcitalopram | LGC | + | 311.2 | 109.111 | 25.118 | 262 | 15.865 | 63 | 8.3 | Citalopram_IS |
| N-desmetyl TRM | LGC | + | 250.34 | 44.222 | 12 | 232.111 | 10 | 55 | 6.3 | Tramadol_IS |
| Norsertraline | LGC | + | 275.122 | 159 | 19.91 | 123 | 42.865 | 57 | 9.5 | Carbamazepine_IS |
| O-Desmethylvenlafaxine | Sigma-Aldrich | + | 264.139 | 58.169 | 18.747 | 246.04 | 10.253 | 47 | 5.9 | Venlafaxine_IS |
| O-desmetyl TRM | Sigma-Aldrich | + | 250.34 | 58.151 | 16 | 232.11 | 10 | 58 | 5.3 | Tramadol_IS |
| Orphenadrine | EDQM | + | 270.19 | 181.04 | 12.225 | 166.054 | 29.416 | 35 | 8.9 | Carbamazepine_IS |
| Oseltamivir | EP | + | 285.16 | 138.071 | 19.354 | 197.071 | 10.253 | 42 | 5.7 | Trimethoprim_IS |
| Oxazepam | Chiron | + | 287.03 | 241 | 22.893 | 269 | 13.691 | 65 | 7.9 | Oxazepam_IS |
| Oxazepam_IS (D5) | Lipomed | + | 292.1 | 246.071 | 23.045 | - | - | 67 | 7.8 | - |
| Oxcarbazepine | Sigma-Aldrich | + | 253.048 | 208 | 20.112 | 180 | 32 | 54 | 6.9 | Carbamazepine_IS |
| Picloram | Sigma-Aldrich | + | 240.939 | 194.96 | 24.36 | 222.889 | 13.742 | 56.202 | 4 | Carbendazim_IS |
| Pirimicarb | Sigma-Aldrich | + | 239.161 | 182.111 | 16.876 | 72.111 | 22.034 | 52.494 | 4.7 | Carbendazim_IS |
| Pirimiphos_ethyl | Sigma-Aldrich | + | 334.161 | 198.111 | 23.045 | 182.111 | 23.399 | 74.247 | 9.9 | Metolachlor_IS |
| Pirimiphos_methyl | Sigma-Aldrich | + | 306.111 | 164.111 | 23.096 | 108.04 | 32.803 | 71.281 | 9 | Metolachlor_IS |
| Pizotifen | Sanza Cruz | + | 296.191 | 96.04 | 22.539 | 199.071 | 26.382 | 72 | 9.2 | Carbamazepine_IS |
| Prometryne | Chem Servise | + | 242.1 | 200.111 | 18.343 | 158.071 | 24.258 | 63.371 | 7 | Atrazine_IS |
| Propachlor | Absolute Standard | + | 212.02 | 169.982 | 15.865 | 94.111 | 28.708 | 46.067 | 7.3 | Simazine_IS |
| Propazine | AccuStandard | + | 230.13 | 188.04 | 17.18 | 146.071 | 22.994 | 71.528 | 7.5 | Simazine_IS |
| Propazine_2-hydroxy | Neochema | + | 212.222 | 170.111 | 18.747 | 128.111 | 23.601 | 61.146 | 5.2 | Carbendazim_IS |
| Propiconazole | Dr. Ehrenstorfer | + | 342.09 | 159.071 | 28.657 | 204.946 | 17.382 | 70 | 8.8 | Metolachlor_IS |
| Propranolol | Sigma-Aldrich | + | 260.117 | 116.111 | 17.938 | 183.071 | 17.938 | 60 | 7.8 | Metoprolol_IS |
| Pyrimethanil | Sigma-Aldrich | + | 200.172 | 107.111 | 25.421 | 183.071 | 25.674 | 73.011 | 6.2 | Simazine_IS |
| Ropinirole | AK Scientific | + | 261.18 | 114.183 | 19.152 | 160.071 | 22.893 | 62 | 5.8 | Metoprolol_IS |
| Rosuvastatin | AK Scientific | + | 482.221 | 258.04 | 33.056 | 270.111 | 36.09 | 101 | 8.6 | Carbamazepine_IS |
| Roxithromycin | Sigma-Aldrich | + | 837.53 | 679.387 | 18.798 | 522.347 | 23.803 | 89 | 9.2 | Clarithromycin_IS |
| Sebuthylazine | AccuStandard | + | 230.1 | 174.111 | 17.787 | 132.111 | 24.056 | 68.562 | 7.7 | Simazine_IS |
| Sertraline | AK Scientific | + | 306.06 | 159 | 28.051 | 274.929 | 10.253 | 37 | 9.7 | Carbamazepine_IS |
| Simazine | Chem Servise | + | 202.019 | 132.058 | 20.213 | 124.111 | 19.303 | 60.157 | 5.9 | Simazine_IS |
| Simazine_hydroxy | Chem Servise | + | 184.1 | 114.111 | 19.809 | 69.058 | 32.702 | 61.64 | 3.3 | Carbendazim_IS |
| Simazine_IS (13C3) | CIL | + | 205.07 | 134.071 | 20.112 | 126.04 | 18.545 | 62.876 | 6 | - |
| Sotalol | AK Scientific | + | 273.1 | 255 | 10.253 | 213 | 18.09 | 48 | 4.5 | Metoprolol_IS |
| Sulfadiazine | Sigma-Aldrich | + | 251.122 | 156 | 15.41 | 108.071 | 24.006 | 54 | 4.4 | Sulfamethoxazole_IS |
| Sulfamerazine | Sigma-Aldrich | + | 265.152 | 172 | 16.27 | 156 | 17.028 | 60 | 4.8 | Sulfamethoxazole_IS |
| Sulfamethazine | HPC | + | 279.1 | 186 | 16.876 | 124.111 | 24.208 | 64 | 5.1 | Sulfamethoxazole_IS |
| Sulfamethizole | Sigma-Aldrich | + | 271.061 | 156 | 14.197 | 108.04 | 23.449 | 52 | 5.4 | Sulfamethoxazole_IS |
| Sulfamethoxazole | Sigma-Aldrich | + | 254.122 | 156 | 16.067 | 108.04 | 24.258 | 55 | 6.2 | Sulfamethoxazole_IS |
| Sulfamethoxazole_IS (D4) | TRC | + | 260.07 | 162.071 | 16.421 | - | - | 55 | 6.2 | - |
| Sulfapyridine | Sigma-Aldrich | + | 250.152 | 156 | 16.27 | 184.071 | 18.242 | 59 | 4.7 | Sulfamethoxazole_IS |
| Tamoxifen | Sigma-Aldrich | + | 372.261 | 72.111 | 23.601 | 129.111 | 26.685 | 78 | 11.5 | Carbamazepine_IS |
| Tebuconazole | AK Scientific | + | 308.161 | 125.071 | 36.949 | 150.982 | 24.764 | 63 | 8.4 | Metolachlor_IS |
| Telmisartan | AK Scientific | + | 515.261 | 276.04 | 46.404 | 497.222 | 32.753 | 127 | 9.5 | Carbamazepine_IS |
| Terbinafine | Sigma-Aldrich | + | 292.2 | 141.04 | 21.831 | 115.04 | 55 | 53 | 10.2 | Carbamazepine_IS |
| Terbutaline | Sigma-Aldrich | + | 226.1 | 152.054 | 16.371 | 125.054 | 24.309 | 46 | 4.4 | Carbamazepine_IS |
| Terbuthylazine | Sigma-Aldrich | + | 230.1 | 174.071 | 18.444 | 104 | 33.966 | 56.202 | 7.8 | Terbuthylazine_IS |
| Terbuthylazine_desethyl | Sigma-Aldrich | + | 202.1 | 146.071 | 15.461 | 104 | 27.039 | 43 | 6.3 | Simazine_IS |
| Terbuthylazine_desethyl-2-hydroxy | Neochema | + | 184.1 | 128.111 | 14.449 | 86.111 | 23.449 | 41 | 2.9 | Carbendazim_IS |
| Terbuthylazine_hydroxy | Sigma-Aldrich | + | 212.13 | 156.111 | 15.258 | 114.111 | 23.702 | 49 | 5.3 | Carbendazim_IS |
| Terbuthylazine_IS |  | + | 235.191 | 179.04 | 17.736 | 137.04 | 25.978 | 56 | 7.7 | - |
| Terbutryn | Chromservice | + | 242.1 | 186.111 | 19.708 | 138.04 | 28.455 | 63.371 | 6.95 | Atrazine_IS |
| Thiamethoxam | Sigma-Aldrich | + | 292.061 | 211.071 | 12.073 | 131.929 | 21.983 | 47.551 | 3.7 | Carbendazim_IS |
| Tramadol | Sigma-Aldrich | + | 264.23 | 58.169 | 16.978 | 246.111 | 10.253 | 46 | 6.3 | Tramadol_IS |
| Tramadol_IS (D3) | Lipomed | + | 267.17 | 58.111 | 17.281 | - | - | 46 | 6.3 | - |
| Trazodone | HPC | + | 372.18 | 148.04 | 33 | 176.04 | 24 | 81 | 7.6 | Carbamazepine_IS |
| Triadimenol | Sigma-Aldrich | + | 296.1 | 227 | 10.253 | 70.111 | 10.506 | 44 | 7.8 | Terbuthylazine_IS |
| Triallat | Sigma-Aldrich | + | 306 | 86.111 | 17.736 | 128.183 | 14.854 | 59.169 | 10.1 | Metolachlor_IS |
| Trimethoprim | Sigma-Aldrich | + | 291.191 | 230.04 | 23.247 | 261.071 | 25.067 | 78 | 5.5 | Trimethoprim_IS |
| Trimethoprim_IS (13C3) | TRC | + | 294.161 | 231.111 | 24.258 | 262.071 | 26.079 | 72.27 | 4.4 | - |
| Triticonazole | Chem Servise | + | 318.13 | 70.111 | 18.646 | 125.071 | 32.348 | 51.258 | 8 | Terbuthylazine_IS |
| Valsartan | Sigma-Aldrich | + | 436.23 | 291.111 | 17.129 | 235.071 | 17.534 | 52 | 9.1 | Carbamazepine_IS |
| Venlafaxine | AK Scientific | + | 278.2 | 58.111 | 19.152 | 260.183 | 10.253 | 49 | 7.2 | Venlafaxine_IS |
| Venlafaxine_IS (D6) | Chiron | + | 284.261 | 266.183 | 10.253 | - | - | 50 | 7.2 | - |
| Verapamil | Sigma-Aldrich | + | 455.352 | 165.111 | 27.14 | 303.183 | 24.461 | 88 | 9.3 | Carbamazepine_IS |
| Vortioxetine | LGC | + | 299.18 | 150.058 | 24 | 256.04 | 20 | 71 | 9.6 | Carbamazepine_IS |
| Warfarin | Sigma-Aldrich | + | 309.1 | 163 | 15.309 | 251 | 20.163 | 50.764 | 7.9 | Terbuthylazine_IS |

^1^ The compounds of Sigma-Aldrich, Fluka, La Roche AG and EDQM (EDQM Reference Standards) were purchased from Sigma-Aldrich spol. s r.o. (Czech Republic), the others (TRC … Toronto Research Chemicals,Canada; CIL … Cambridge Isotope Laboratories, Inc., USA; CND Isotopes … C/D/N Isotopes Inc., Canada; Lipomed AG, Switzerland; Alsa Chim, France; AK Scientific, USA; Chemos GmbH, Germany; Chiron Chemicals, Australia; Santa Cruz Biot, USA) were purchased via Labicom s.r.o. (Czech Republic).

SM2 Table S2. Information about analytical methods

HPLC-ESI-MS/MS method for target analysis

-duration 10 min., selected reaction monitoring (SRM) acquisition mode

-mobile phase A: ultra-pure water with 0.1 % formic acid; mobile phase B: acetonitrile with 0.1 % formic acid

LC gradient:

| Time [min] | Mobile phase A [%] | Mobile phase B [%] | Flow [µL/min] |
| --- | --- | --- | --- |
| 0 | 100 | 0 | 300 |
| 1 | 100 | 0 | 300 |
| 5 | 70 | 30 | 350 |
| 7 | 0 | 100 | 400 |
| 8 | 0 | 100 | 400 |
| 8.01 | 100 | 0 | 300 |
| 10 | 100 | 0 | 300 |

HPLC-ESI-HRMS method for nontarget full scan analysis

-duration 15 min., data independent acquisition mode

-mobile phase A: ultra-pure water with 0.1 % formic acid and 0.1mMol Ammonium acetate; mobile phase B: acetonitrile with 0.1 % formic acid and 0.1 mMol Ammonium acetate

LC gradient:

| Time [min] | Mobile phase A [%] | Mobile phase B [%] | Flow [µL/min] |
| --- | --- | --- | --- |
| 0 | 95 | 5 | 300 |
| 1 | 95 | 5 | 300 |
| 4 | 85 | 15 | 300 |
| 8 | 50 | 50 | 350 |
| 10 | 0 | 100 | 350 |
| 12 | 0 | 100 | 350 |
| 12.05 | 95 | 5 | 300 |
| 15 | 950 | 5 | 300 |

SM2 Table S3. Settings of CompoundDiscoverer Workflow for positive and negative mode datasets

| **Parameter** | **ESI+** | **ESI-** |
| --- | --- | --- |
| Min Precursor Mass | 61 Da | 61 Da |
| Max. Precursor Mass | 900Da | 900Da |
| Total Intensity Threshold | 2000 | 2000 |
| Detect Compounds - S/N Threshold | 10 | 10 |
| Detect Compounds -Mass Tolerance | 5ppm | 5ppm |
| Detect Compounds -Min. Peak Intensity | 10 000 | 10 000 |
| Isotope Patterns Mass Tolerance | 5 ppm | 5 ppm |
| predict Compositions - Mass Tolerance | 5 ppm | 5 ppm |

**Filters used on datasets from CD 3.1 for obtaining a final compounds list and numbers of compounds**

|  | **ESI+ compounds** | **ESI- compounds** |
| --- | --- | --- |
| merged features | 22 931 | 9 052 |
| all compounds | 5 463 | 2 513 |
| background | 4 570 | 2 426 |
| background is false | 1 607 | 722 |
| Areas greater than 50 000 | 2 963 | 1 704 |
| RT greater that 1 min | 2 963 | 1 704 |
| Formula is not blank | 2 961 | 1 704 |
| **Compound’s list** | **2 881** | **1 689** |

SM2 Table S4. Values of isotope standards relative standard deviation for peaks areas and retention time.

| **Compound** | **Ionization mode** | **Area** | | | | **Retention time (min)** | | | |
| --- | --- | --- | --- | --- | --- | --- | --- | --- | --- |
|  |  | **%RSD** | max | min | average | **%RSD** | max | min | average |
|  |  |  |  |  |  |  |  |  |  |
| Amitriptyline | positive | **12.98** | 4.0E+07 | 2.3E+07 | 3.5E+07 | **0.31** | 9.5 | 9.4 | 9.5 |
| Atenolol | positive | **5.06** | 1.3E+07 | 1.1E+07 | 1.2E+07 | **5.7** | 2.1 | 1.8 | 1.9 |
| Caffeine | positive | **10.42** | 9.8E+06 | 6.5E+06 | 8.3E+06 | **0.34** | 5.5 | 5.4 | 5.4 |
| Carbamazepine | positive | **10.64** | 4.6E+07 | 3.1E+07 | 3.7E+07 | **0.3** | 9.1 | 9.0 | 9.0 |
| Citalopram | positive | **7.53** | 3.2E+07 | 2.5E+07 | 2.9E+07 | **0.34** | 8.5 | 8.4 | 8.4 |
| Clarithromycin | positive | **16** | 6.2E+06 | 3.7E+06 | 4.8E+06 | **0.21** | 10.0 | 9.9 | 9.9 |
| Clindamycin | positive | **4.39** | 2.0E+07 | 1.7E+07 | 1.8E+07 | **0.34** | 8.3 | 8.3 | 8.3 |
| Diclofenac | positive | **11.21** | 3.0E+06 | 2.1E+06 | 2.6E+06 | **0.26** | 10.4 | 10.3 | 10.4 |
| Metoprolol | positive | **6.48** | 3.3E+07 | 2.5E+07 | 2.9E+07 | **0.48** | 6.6 | 6.5 | 6.5 |
| Oxazepam | positive | **6.51** | 3.0E+06 | 2.4E+06 | 2.7E+06 | **0.34** | 9.6 | 9.5 | 9.5 |
| Sulfamethoxazole | positive | **7.61** | 1.9E+07 | 1.5E+07 | 1.8E+07 | **1.65** | 6.3 | 6.0 | 6.2 |
| Tramadol | positive | **4.09** | 3.2E+07 | 2.9E+07 | 3.0E+07 | **0.43** | 6.2 | 6.2 | 6.2 |
| Trimethoprim | positive | **7.9** | 3.5E+07 | 2.8E+07 | 3.2E+07 | **0.66** | 5.4 | 5.3 | 5.3 |
| Venlafaxine | positive | **5.29** | 3.4E+07 | 2.9E+07 | 3.2E+07 | **0.38** | 7.7 | 7.6 | 7.7 |
| Metazachlor ESA | negative | **10.72** | 1.8E+05 | 1.3E+05 | 1.5E+05 | **0.42** | 7.5 | 7.4 | 7.4 |
| Oxazepam | negative | **17.43** | 5.1E+04 | 3.1E+04 | 4.0E+04 | **0.83** | 9.7 | 9.4 | 9.6 |
| Diclofenac | negative | **13.12** | 6.0E+05 | 3.8E+05 | 4.7E+05 | **0.3** | 10.5 | 10.3 | 10.4 |
| 2,4-Dichlorophenoxyacetic acid | negative | **14.62** | 3.4E+05 | 2.0E+05 | 2.8E+05 | **0.38** | 8.9 | 8.8 | 8.9 |
